# The influence of microbiota on ferroptosis in intestinal diseases

**DOI:** 10.1080/19490976.2023.2263210

**Published:** 2023-10-05

**Authors:** Ting Yao, Lanjuan Li

**Affiliations:** State Key Laboratory for Diagnosis and Treatment of Infectious Diseases, National Clinical Research Center for Infectious Diseases, National Medical Center for Infectious Diseases, Collaborative Innovation Center for Diagnosis and Treatment of Infectious Diseases, The First Affiliated Hospital, Zhejiang University School of Medicine, Hangzhou City, China

**Keywords:** Ferroptosis, microbiota, intestinal diseases, oxygen stress, lipid peroxidation

## Abstract

Ferroptosis is a distinctive form of iron-dependent necrotic cell death, characterized by excessive lipid peroxidation on cellular membranes and compromised cellular antioxidant defenses. Multiple metabolic pathways, including iron and lipid metabolism, as well as antioxidant systems, contribute to the execution of ferroptosis. The gut microbiota exerts regulatory effects on ferroptosis through its microbial composition, biological functions, and metabolites. Notably, most pathogenic bacteria tend to promote ferroptosis, thereby inducing or exacerbating diseases, while most probiotics have been shown to protect against cell death. Given microbiota colonization in the gut, an intimate association is found between intestinal diseases and microbiota. This review consolidates the essential aspects of ferroptotic processes, emphasizing key molecules and delineating the intricate interplay between gut microbiota and ferroptosis. Moreover, this review underscores the potential utility of gut microbiota modulation in regulating ferroptosis for the treatment of intestinal diseases.

## Introduction

Ferroptosis is considered a novel type of inflammatory cell death different from apoptotic, necroptotic, pyroptotic, and autophagic forms of cell death, featured by iron overload and unrestrained lipid peroxidation accumulation.^1,2^ Increasing researches indicate that ferroptosis plays a vital role in the progression of nonalcoholic fatty liver disease (NAFLD),^3^ inflammatory bowel disease (IBD),^4^ neurodegenerative disorders,^5,6^ and various cancers.^4,7^ Although “ferroptosis” was first defined in 2012,^8^ the fields of iron metabolism, reactive oxygen stress (ROS), and lipid peroxidation have been explored for a long time, even dating back to the earliest research on cystine and glutathione (GSH) in the 1950s.^1^ Ferroptosis occurs as a consequence of disorder in diverse metabolic processes and signaling pathways, such as GSH synthesis, ROS accumulation, lipid peroxidation, cysteine transport,^1,9^ communication with endoplasmic reticulum pressure and autophagy.^10,11^ Unique morphological features of ferroptosis include smaller mitochondria, the decrease or vanishment of mitochondrial cristae, and an increased density of mitochondrial membrane with the rupture of cell membrane.^12^

Intestinal microecology has recently gained widespread attention due to its ability to cause or alleviate diseases. Trillions of commensal microorganisms colonize the mammalian intestine and interact with the host in physiological processes, such as cell proliferation and immune responses, and even co-evolve with their hosts over millennia.^13–15^ Besides the well-known pathogenic microorganisms that cause various severe infectious diseases, such as *Shigella* species,^16^ there are numerous symbiotic microbes, such as *Lactobacillus* and *Bifidobacterium*, that regulate host physiological activities including digestion, cognitive development, metal element intake, immune homeostasis, epithelial barrier function and restrict pathogens.^13,17–19^ “Dysbiosis”, which means alteration in the microbiota composition including changes in bacterial abundance, loss of beneficial bacteria, and increased pathogen levels, have been observed to be associated with multiple diseases, especially in intestinal diseases.^13,20^Furthermore, metabolites derived from gut microbe, encompassing short-chain fatty acids (SCFAs), bile acids (BAs), and neurotransmitters, regulate the processes of remote organs. These intricate interrelationships are commonly referred to as the gut-brain axis, gut-kidney axis, gut-liver axis, and gut-skin axis.^21,22^

The gut microbiota’s ability to regulate ferroptosis has garnered attention. These microbes influenced the level of hepcidin, which was a primary regulator of iron homeostasis synthesized by the liver.^23^ Other researches have demonstrated that disturbed gut microbiota induced ferroptosis within the gastrointestinal tract, whereas the supplementation of probiotics exerted an inhibitory effect on ferroptosis by preventing iron overload and lipid peroxidation.^24,25^ The potential therapeutic application of inhibiting ferroptosis appears promising for the management of degenerative or chronic inflammatory diseases. However, in the context of cancer treatment, the induction of ferroptosis becomes imperative. Based on recent studies, most pathogenic bacteria facilitate ferroptosis to aggravate cell loss and inflammation, while most probiotics alleviate ferroptosis.

This review begins by introducing the critical molecules and periods involved in ferroptosis. It then focuses on the role of the gut microbiota in regulating host ferroptosis and the underlying mechanisms. Finally, it discusses how gut microbiota influences the progression of intestinal diseases by modulating ferroptosis. The aim is to provide alternative strategies that target the gut microbiota and ferroptosis for more effective treatment of intestinal diseases in the future.

## Iron cycle and metabolism

Iron functions as an important component of hemoglobin, myoglobin, cytochromes, catalases, peroxidases and metalloenzymes, and participates in numerous biochemical processes, including oxygen transport, energy production, immune regulation, DNA synthesis.^26^ Deficiency of iron usually causes anemia, leading to adverse consequences such as a higher risk of maternal and child mortality, impaired cognition, reduced physical performance and a lower quality of life.^27^ Therefore, individuals with iron-deficiency anemia are advised to take daily iron supplements. However, excessive iron creates an environment conducive to ferroptosis, resulting in damage of DNA, protein, and lipids.^27,28^

**Figure 1.**
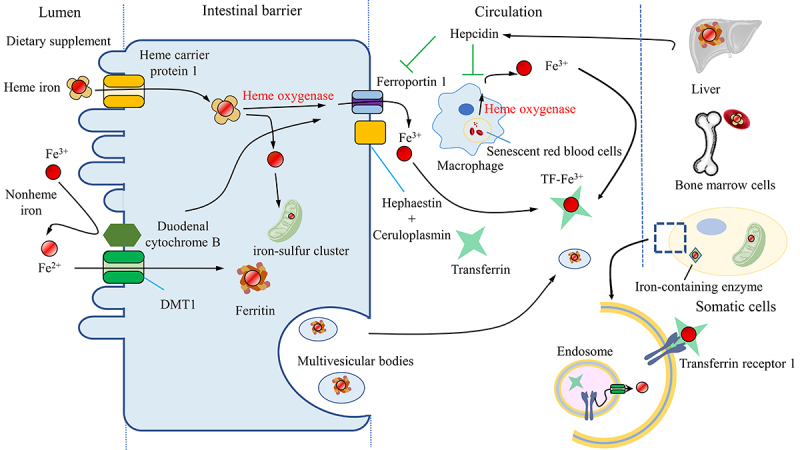
The iron cycle and metabolism. Iron is obtained from the diet in the form of heme or nonheme iron and is absorbed by enterocytes while Fe^3+^ is reduced to Fe^2+^ by reductase before being absorbed by the receptor DMT1. Macrophages phagocytose senescent blood cells, degrade heme via heme oxygenase and release free iron. The absorbed iron is then either stored in ferritin, used for the biosynthesis of iron-sulfur clusters, or exported by ferroportin1 with a multi-copper ferroxidase such as ceruloplasmin, which oxidizes Fe^2+^ to Fe^3+^ in the plasma membrane. Iron can also be exported with ferritin by multivesicular bodies. Iron in the blood is bound to transferrin, forming an iron-transferrin complex. This complex is transported to the liver for storage, to bone marrow cells for heme synthesis, and to other tissues for the synthesis of iron-containing enzymes. Cells take up iron through transferrin receptor 1-mediated endocytosis. Fe^3+^ is liberated from transferrin and subsequently reduced to Fe^2+^ by endosomal reductases. Hepcidin, secreted by the liver, either inhibits the degradation of ferroportin1 or directly blocks the channel, allowing iron to accumulate within the cell. DMT1, divalent metal-ion transporter-1.

An integrated and precise system, monitoring the process of iron uptake, storage, utilization, and release orderly, controls the cellular level of free iron strictly. Iron in body mainly comes from senescent red blood cells recycled by reticuloendothelial macrophages and dietary supplement absorbed by enterocytes.^29^ Iron in the diet is primarily present as ferric iron (Fe^3+^), which has limited solubility and bioavailability. To increase absorption, duodenal cytochrome B (DCYTB), an iron-reducing ferric reductase found on the apical membrane of enterocytes, reduces Fe^3+^ to ferrous iron (Fe^2+^), which is then absorbed via divalent metal-ion transporter-1 (DMT1).^27,30^ Moreover, a portion of dietary iron is heme iron found in meat, which is absorbed through heme carrier protein 1 and then degraded to free Fe^3+^ by heme oxygenase (HO) in enterocytes.^31^ After absorption by enterocytes, iron is transported to the portal blood by the Fe^2+^ exporter, ferroportin 1 (FPN1), working in concert with a multi-copper ferroxidase such as ceruloplasmin to oxidize Fe^2+^ to Fe^3+^ in the plasma membrane.^32^ In the bloodstream, Fe^3+^ bound by transferrin (Tf) is taken up by hepatocytes, macrophages, bone marrow cells and other cells via transferrin receptor 1 (TfR1) mediated endocytosis.^33,34^ Upon entering the cell, Fe^3+^ is liberated from Tf and subsequently reduced to Fe^2+^ by endosome reductases, such as six-transmembrane epithelial antigen of the prostate. The Fe^2+^ is then transported out of the endosome and into the cytosol via DMT1.^30^ Cellular iron is either directed to mitochondria for the biosynthesis of iron-sulfur clusters or stored as ferritin or used in the generation of heme in the bone marrow.^27,30^ Moreover, multivesicular bodies and exosomes that carry ferritin excrete iron to decrease intracellular iron levels; and autophagy-dependent degradation of the ferritin also plays a role in maintaining iron balance in the body.^35^ (Figure 1).

### Key molecules of iron metabolism

The disturbance of iron metabolism is a high-risk factor in the early stages of ferroptosis. Interfering with key elements of iron metabolism at both the transcriptional and post-transcriptional levels will disrupt the homeostasis of iron in body.

#### Ferritin

Ferritin binds to approximately 4500 Fe^3+^ and forms a significant iron reservoir, reducing the toxicity of iron in the cytoplasm.^8,36^ Serum ferritin acts as a biomarker to reflect the iron level in patients clinically, and its expression is also affected by storage of iron and inflammatory condition.^37^ The liver is an organ responsible for maintaining the balance of iron exposure because it not only receives substances absorbed by the intestine via the hepatic portal circulation,^38^ but also synthesizes a high amount of ferritin to store and Tf to transport iron.^33^ Existing researches show that diminishing ferritin expression enriches the intracellular labile iron pool (LIP), one redox-active component of non-transferrin bound iron, and even increased iron absorption in enterocyte,^39^ thus causing a toxic high iron status and increased sensitivity to ferroptosis.^28,40^ Moreover, the iron in the LIP predominantly binds to GSH, which can easily become free.^41^ Ferritin is regulated by a variety of molecules at the transcriptional level, such as tumor necrosis factor (TNF), cyclic adenosine monophosphate (cAMP), c-myc as well as at the posttranscriptional level, such as nitric oxide, superoxide, hydroxyl radicals.^40^ In addition, ferritinophagy, which involves degrading ferritin via autophagy, is positively related to ferroptosis, with the evidence that the inhibition of autophagy-related genes reduces LIP, making cells resistant to ferroptosis.^42^ Therefore, ferritin is proposed to be one of the vital and indicative molecules in ferroptosis, as it chelates excessive free iron and adjusts cellular iron content to protect cells from iron toxicity.^43^

#### Transferrin (Tf) and transferrin receptor (TfR)

Iron bound to Tf is the most common form in blood circulation. Serum Tf exists in several forms, such as non-iron bound (apo-Tf), monoferric or diferric (Tf carrying two Fe^3+^) forms.^34^ TfR is a predominant transmembrane glycoprotein that mediates cellular iron uptake via endocytosis of iron-Tf complex in blood circulation and controls cellular iron at an appropriate concentration.^44^ TfR1 is the primary receptor responsible for Tf transport in vertebrates.^45^ Additionally, TfR2, a paralog of TfR1, is only identified on hepatocytes and erythroid precursors as a sensor rather than a transporter of iron.^46^ If the binding capacity of Tf is exceeded or if there is a systemic iron overload, iron will bind loosely to albumin or low-molecular-weight molecules, which is easy to catalyze Fenton and Haber-Weiss reactions to enhance ferroptosis.^29^

#### Ferroportin (FPN) and hepcidin

Ferroportin, a multiple transmembrane protein found on various cell types, primarily on the basolateral membrane of enterocytes and macrophage, functions as a unique exporter for transferring iron from cell to internal environment.^32^ FPN is accompanied by oxidation ability of ferroxidases since it transforms cellular Fe^3+^ to Fe^2+^. Hepcidin is generated and secreted into the bloodstream by hepatocytes. It binds to FPN on cell surfaces, then ubiquitinates, internalizes, and ultimately degrades FPN, or directly blocks the channel to allow iron to accumulate within the cell.^47,48^ There is a negative feedback response between hepcidin synthesis and serum iron concentrations, which helps to prevent potential toxic iron accumulation.^49^ (Figure 1).

## Intestinal microorganisms act on ferroptosis

Recent researches have highlighted the association between the microbiota and cellular ferroptosis, although most studies have been speculative and lack direct evidence. Considering the mechanism of ferroptosis, which includes iron accumulation, ROS production, fatty acid supply and lipid peroxidation, probiotics may inhibit ferroptosis by chelating metal ion, suppressing ROS production or accelerating their clearance, and suppressing key enzymic reduction function.^50^ Furthermore, intestinal microbiota regulates ferroptosis either through direct contact or via metabolites such as BAs, SCFAs, and neurotransmitters.^51^ Nevertheless, it is important to note that the accumulation of ROS alone does not necessarily lead to ferroptosis, as ROS overload is also observed in other cellular processes, such as apoptosis. Similarly, lipid peroxidation can occur independently of iron under conditions of high oxygen pressure. Table 1 summarizes the findings of studies that have identified specific bacteria or their metabolites and their influence on ferroptosis.

**Table 1.** The compositions or metabolites of microbiota affect ferroptosis.

Microbiota	The function to the ferroptosis	Ref.
Lipopolysaccharides	Activation of ACSL4 by up-regulating special protein 1. Regulation of the secretion of serum ferritin. Aggravation of lipid metabolic disorders and ferroptosis in hepatocytes.	^52,53^
Glycochenodeoxycholate	Promotion of TFR-ACSL4-mediated ferroptosis	^38^
Short-chain fatty acid	Facilitation of mitochondrial Ca2+ and GPX4-dependent ferroptosis. Inhibition of cystine/glutamate transporter system by the upstream molecular RBM3 or FFAR2-AKT-NRF2 axis and FFAR2-mTORC1 axis and c-Fos.	^54–56^ ^57–59^
	Reduction of the production of ROS, enhancing oxidative phosphorylation and β-oxidation in physiological conditions. Activation of the PGC1α signaling axis to promote mitochondrial biogenesis; Protection of mitochondria.	
Indigenous bacteria (metabolites, reuterin and 1,3, diaminopropane)	Suppression of HIF-2α, the master transcription factor of intestinal iron absorption and transportation Increase in the iron storage protein ferritin.	^60^
Capsiate	Promotion of Gpx4 expression and restraint of ferroptosis via the overexpression of TRPV1 in intestinal I/R injury.	^61^
Urolithins	Increase in mitophagy and mitochondrial function by reducing excessive inflammation.	^62^
5-HT and 3-HA	Elimination of radicals to resist ferroptosis	^63^
Histamine	Histamine deficiency accelerates myocardial ferroptosis by repressing the activation of STAT3, accompanied by decreased expression of SLC7A11, a major modulator of ferroptosis.	^64^
*Lactobacillus rhamnosus GG*	Regulation of lipid metabolism to inhibit ferroptosis	^24^
*Aeromonas hydrophila*	Increase in the levels of MDA and Fe^2+^ in brain tissues and decrease in GSH.	^65^
*Pseudomonas aeruginosa*	Elevation of levels of oxidized AA-phospholipids by expressing pLoxA (its mammalian orthologue is ALOX15).	^66^
*Mycobacterium tuberculosis*	Reduction in levels of GSH and Gpx4, along with increased levels of free iron, mitochondrial superoxide, and lipid peroxidation; alleviation of the disease is suppressed by Ferrostatin-1.	^67^
*Porphyromonas gingivalis*	Increase in ACSL4, Ptgs2, and Ncoa4 expression, while decreasing GPX4 and SLC7A11.	^68^
*Lactiplantibacillus plantarum*	Transformation of unsaturated fatty acids to resist ferroptosis and their derivatives promote antioxidative gene expression.	^69^
*Escherichia coli*	Increase in intracellular iron levels by inhibiting the expression of Ferroportin-1, followed by the induction of the Fenton reaction to release ROS.	^70^
*Edwardsiella piscicida*	Promotion of iron accumulation, mitochondrial dysfunction, and production of ROS	^71^

HIF-2α, Hypoxia inducible factor 2α; ACSL4, Acyl-CoA synthetase long-chain family 4; GPX4, glutathione peroxidase 4; RBM3, RNA-binding motif protein 3; FFAR2, Free fatty acid receptor 2; NRF2, Nuclear factor erythroid 2-related factor 2; mTORC1, mTORC1, mammalian target of rapamycin complex 1; TRPV1, transient receptor potential vanilloid 1; I/R, Ischemia-reperfusion; TFR, transferrin receptor; 5-HT, serotonin; STAT3, Signal Transducer and Activator of Transcription 3; SLC7A11, solute carrier family 7 member 11; MDA, Malondialdehyde; GSH, glutathione; AA, arachidonic acid; ALOX15, arachidonate 15-lipoxygenase; Ptgs2, prostaglandin-endoperoxide synthase 2; Ncoa4, Nuclear Receptor Coactivator 4; RBCs, Red blood cells; ROS, reactive oxygen species.

### Iron accumulation

According to the theory, alterations in iron metabolism resulting in iron level fluctuations could potentially influence the occurrence of ferroptosis. However, limited literature has indicated the impact of the microbiota on ferroptosis via modulation of iron metabolism, despite numerous investigations demonstrating the ability of the microbiota to modulate host iron homeostasis.^60,72^ Excess iron, as a redox-active toxicant, initiates excessive ROS generation and destroys cellular structure though Fenton and Haber-Weiss reaction.^73^ Firstly, the microbiota modulates the expression of key molecules involved in iron metabolism. Gut microbiota regulated plasma ferritin levels and iron concentration in esophageal tissue;^52^ and their metabolites were observed to suppress hypoxia-inducible factor 2α (HIF-2α) to modulate intestinal iron absorption and increase the ferritin levels.^60^ Additionally, microbial stimulation promoted the expression of hepcidin.^74^

To reduce the toxic effects of excessive free iron, reducing the supply of an iron agent seems to be a feasible method. However, iron supplementation is the common method for treating anemia. Even worse, in IBD with anemia, supplementing with iron may also lead to exacerbate colon symptoms. Fortunately, probiotics offer a solution to this problem.^75^ It has been shown that taking FeSO_4_ with probiotics, such as *Lactobacillus alimentarius NKU556*, is not only more effective in improving iron deficiency in the body but also results in fewer side effects in the intestine.^76^ Another method is to increase the utilization rate of existing iron in the intestine and eliminate additional iron supplementation under physiological conditions, for a large portion of iron in the intestine is not utilized.^46^ A recent study demonstrated that certain probiotics, such as *Bifidobacteria* and *Lactobacillus*, promoted iron absorption by forming essential amino acids or SCFAs to reduce intestinal pH to optimize dietary iron bioavailability without imposing additional burdens from iron supplements on the gastrointestinal tract.^77^

The microbiota in the human gastrointestinal tract competes with host for iron utilization for their own various physiological activity via expression of the FeoB protein, siderophores (the metal-chelating agents to seize the insoluble ferric iron and chelate iron),^78^ enterobactin, and other related proteins.^78,79^ Some researchers believe that commensal bacteria share iron to host,^46^ transform inorganic iron to organic forms to low toxic of free iron,^80^ and prevent the outgrowth of potentially harmful microbes through competition for iron.^81^ The optimal condition is for both the host and commensal bacteria to acquire enough iron for their life processes without experiencing iron-induced death due to excess iron.

**Figure 2.**
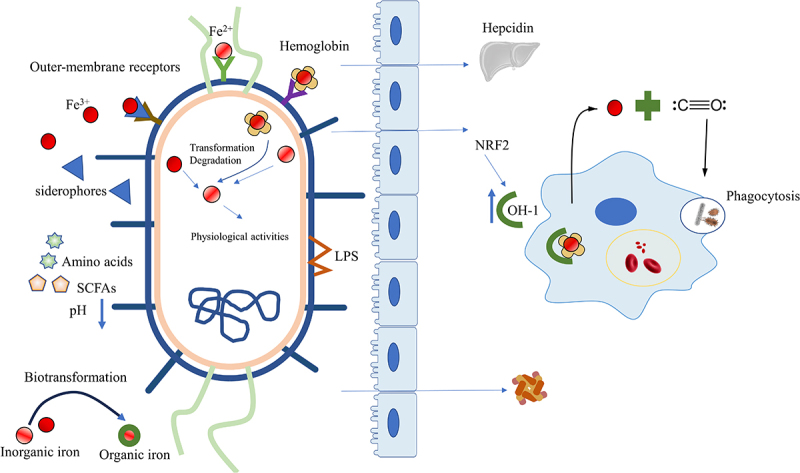
Intestinal microbiota affects iron metabolism. Microorganisms utilize various mechanisms to obtain different forms of iron (Fe^3+^, Fe^2+^, heme iron). Moreover, the microbiota reduces intestinal pH by producing essential amino acids or SCFAs to optimize dietary iron bioavailability, regulates the expression of hepcidin, transforms inorganic iron into organic forms to lower the toxicity of free iron and modulates the ferritin levels. The microbiota also activates the HO-1/CO pathway or NRF2/HO-1 to strengthen phagocytosis and increase macrophage processing of hemoglobin for iron release. SCFAs, Short-chain fatty acids; HO-1, heme oxygenase-1; CO, carbon monoxide; NRF2, Nuclear factor erythroid 2-related factor 2.

The phagocytosis of senescent red blood cells by macrophages and the subsequent liberation of iron from heme constitute the primary source of iron in the body, alongside dietary intake. Heme oxygenase-1 (HO-1) plays a critical role in regulating intracellular iron concentrations and oxidative stress by catalyzing heme into Fe^2+^, carbon monoxide (CO), and biliverdin/bilirubin.^82^ Notably, post-colonization with microbiota induced significant expression of HO-1 in wild-type mice but not in germ-free controls, highlighting the critical role of microbiota in regulating HO-1 expression.^83,84^ HO-1 is upregulated in response to oxidative stress to protect host cells in general. During infections, HO-1 is upregulated to strengthen phagocytosis and promote bacterial clearance.^84^ Probiotics activate the nuclear factor erythroid 2-related factor 2 (NRF2)/HO-1 pathway to alleviate diseases: *L. acidophilus ATCC 4356* for renal ischemia-reperfusion injury (IRI);^85^ *Bacillus coagulans JA845* for neurodegenerative disorders,^86^ *Lactobacillus* for oxidative stress-related intestinal disease^87^ and nonalcoholic steatohepatitis.^88^ Nevertheless, elevated HO-1 during *Mycobacterium tuberculosis* (MTB) infection not only enhances iron availability, promoting MTB survival, but also raises intracellular iron levels and ROS production, subsequently leading to lipid peroxidation and ferroptosis.^89^ (Figure 2).

### Oxidative stress

The induction of oxidative stress can be attributed to the iron-dependent Fenton reaction, as well as to enzymes belonging to the nicotinamide adenine dinucleotide phosphate (NADPH) oxidase (NOX) family and mitochondria.^73^ The Fenton reaction involves the reaction of Fe^2+^ and hydrogen peroxide (H_2_O_2_) to produce Fe^3+^, OH‐, and the hydroxyl radical (OH∙), which is one of the most aggressive forms of ROS.^90^ Subsequently, the Haber-Weiss cycle occurs with O2·− reacting with Fe^3+^ to regenerate Fe^2+^ and OH∙, leading to peroxidation of almost all substances, including lipids and proteins, ultimately resulting in disrupting cell membranes. Interestingly, studies have shown that *Escherichia coli* has developed mechanisms to remove substrates of the Fenton reaction, assimilate Fe^2+^, and decompose H_2_O_2_ using enzymes synthesized by the bacteria themselves, which may apply to attenuate the inflammatory responses.^91,92^

The role of mitochondria in ferroptosis remains a topic of debate, despite evidence that impaired mitochondria produce an elevated amount of ROS during oxidative phosphorylation.^1^ Some studies have suggested that cells knockout of mitochondrial DNA remain susceptible to ferroptosis,^93^ while other studies have indicated that mitochondria are crucial for inducing ferroptosis in response to erastin or cystine starvation, but without a significant role in RSL3-induced ferroptosis.^94^ Nevertheless, mitochondrial dysfunction is associated with weakened antioxidant systems and an increased reliance on glutaminolysis-driven tricarboxylic acid (TCA) cycles.^95^ Recent research has shown that probiotics have a protective effect on mitochondria.^54,96^ For instance, butyrate in cell lines derived from patients with autism has been found to regulate mitochondrial functions, including enhancing oxidative phosphorylation and β-oxidation under physiological stress or mitochondrial dysfunction^54^ and activating the peroxisome proliferator-activated receptor-gamma coactivator-1alpha (PGC1α) signaling axis in NSC34-G93A cells to regulate mitochondrial biogenesis.^55^ However, another study suggested that mitochondrial function and glutathione peroxidase 4 (GPX4)-dependent ferroptosis were boosted under the condition of supplementing butyrate.^56^ We speculate that the occurrence of this phenomenon may be dose-related. Furthermore, another metabolite of gut bacteria, urolithins, enhances cellular health by increasing mitophagy and mitochondrial function and by reducing excessive inflammation.^62^

**Figure 3.**
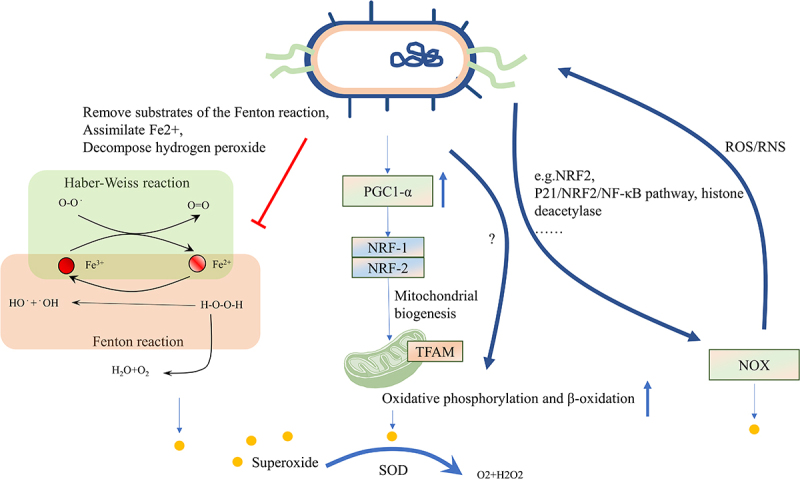
Intestinal microbiota affects oxidative stress. The induction of oxidative stress can be attributed to the iron-dependent Fenton reaction, as well as enzymes belonging to the NOX family and mitochondria. Microbes can remove substrates of the Fenton reaction, assimilate Fe^2+^, and decompose H_2_O_2_ using enzymes synthesized by the bacteria themselves. Microbes can also enhance mitochondrial functions, including oxidative phosphorylation and β-oxidation, under physiological stress or mitochondrial dysfunction by activating the PGC1α signaling pathway. Microbes can increase NOX1 levels and activate the NRF2 pathway, while suppressing histone deacetylase activity to prevent ROS formation. Additionally, SOD in microbes can reduce ROS levels in the host. The level of ROS can also affect the microbes themselves. NOX, nicotinamide adenine dinucleotide phosphate oxidase; H_2_O_2,_ Hydrogen peroxide; PGC1α, peroxisome proliferator-activated receptor-gamma coactivator-1alpha; NRF2, Nuclear factor erythroid 2-related factor 2; ROS, reactive oxygen stress; SOD, superoxide dismutases.

NOX, the enzyme related to ROS accumulation, deliberately generates superoxide anion to maintain homeostasis and fight microorganisms that penetrate the mucus layer in the epithelial cells. NOX is implicated in the initial generation of lipid ROS during ferroptosis. Inhibition of NOX hindered ferroptosis in both human and plant cells.^97,98^ Two key members of the NOX family, NOX1 and dual oxidase 2 (DUOX2), have been identified as crucial mediators that link gut bacteria and the host. NOX1 produces superoxide, while DUOX2 generates H_2_O_2_.^18^ The evidence shows that NOX modulates the colonic microbe in healthy and 1% dextran sulfate sodium (DSS)-induced low-grade inflammation mice model by producing ROS/RNS.^99^ Transient enzymatic production of ROS by NOX1 and NOX2 within enterocytes occurs in response to specific taxa of intestinal bacteria.^15^ *Lactobacillus rhamnosus GG (LGG)* has been not only been proven to increase NOX1 and activate the NRF2 pathway, but also to contribute to restoring the intestine microbiota, facilitating epithelial cell proliferation.^100^ Supplying mice with segmented filamentous bacteria increases the expression of DUOX2 in the intestine, while mucosal dysbiosis causes increased expression of DUOX2 independent of interleukin (IL)17 or IL22.^101^ Butyrate has been shown to activate P21/NRF2/NF-κB pathway to prevent the accumulation of ROS by inhibiting the expression of NOX2 and increasing superoxide dismutases (SOD).^102^ During the process of acute kidney injury, acetate suppressed histone deacetylase activity, weakening the NOX2/ROS signaling in T cells.^103^ It’s worth noting that microorganisms have developed a battery of enzymes to detoxify of ROS, which encode SOD, catalases, thioredoxin and a thioredoxin reductase, as well as glutathione system. The microbial antioxidant system protects microorganisms from engulfment in phagocytic cells, which is highly unfavorable for infection control.^78^ Nevertheless, whether the ability of probiotics to remove ROS could be used to reduce ferroptosis needs further exploration. One study showed that *Lactobacillus gasseri* with inserted manganese SOD was more effective in alleviating inflammation compared to *Lactobacillus gasseri* without SOD in IBD mice model.^104^ (Figure 3).

### Fatty acid

Indeed, polyunsaturated fatty acids (PUFAs), particularly long-chain PUFAs containing multiple double bonds, such as arachidonic acid (AA), linoleic acid (LA) and docosahexaenoic acid (DHA), are more susceptible to oxidation than monounsaturated and saturated fatty acids. The pentadiene structure of the PUFAs is particularly vulnerable to oxidation^73^ while omega-6 PUFAs (*n*-6 PUFAs), but not *n*-3 PUFAs (considering similar chain length and degree of saturation), are more sensitize to oxidative stress. Moreover, 7-dehydrocholesterol, which is a precursor to cholesterol, exhibits significantly higher redox activity compared to cholesterol and AA.^105^ The susceptibility of cells to ferroptosis could manipulate the synthesis or degradation process of PUFAs. Nevertheless, the relationship between lipid metabolism and ferroptosis is intricate and debatable.^106^ For one thing, ferroptosis-induced lipid ROS increases the formation of lipid droplets,^3^ PUFAs serve as the substrates for lipid peroxidation,^107^ and excessive synthesis of fatty acids in the body or intake from the outside promoted ferroptosis. On the other hand, fatty acid β-oxidation hinders ferroptosis by reducing the abundance of unesterified PUFAs.^106^ Therefore, modifying lipid metabolism, with a particular focus on PUFAs, represents a potential strategy for modulating ferroptosis.^108,109^

Gut microbiota influences ferroptosis by directly or indirectly regulating the absorption, bioavailability, and biotransformation of PUFAs.^110^ Some specific strains of microbiota, such as lactic acid-producing bacteria, are known to mitigate the toxicity of PUFAs by mediating saturation and producing PUFAs-derived intermediate metabolites.^111^ *Lactiplantibacillus plantarum* converts LA to conjugated LA and oleic acid through multi-enzymatic reactions in the host gastrointestinal tract. Additionally, the efficient production of intermediates, 10-Oxo-trans-11-octadecenoic acid, activated the NRF2-antioxidant response element (ARE) pathway to promote antioxidative gene expression.^69^ SCFAs generated by gut microbes influenced the metabolism and absorption of omega-3 PUFAs. In turn, in animal, supplementation with omega-3 PUFAs has been found to increase the abundance of several SCFA-producing bacteria, including *Bifidobacterium*, *Roseburia*, and *Lactobacillus* in the mouse intestinal tract.^112^

**Figure 4.**
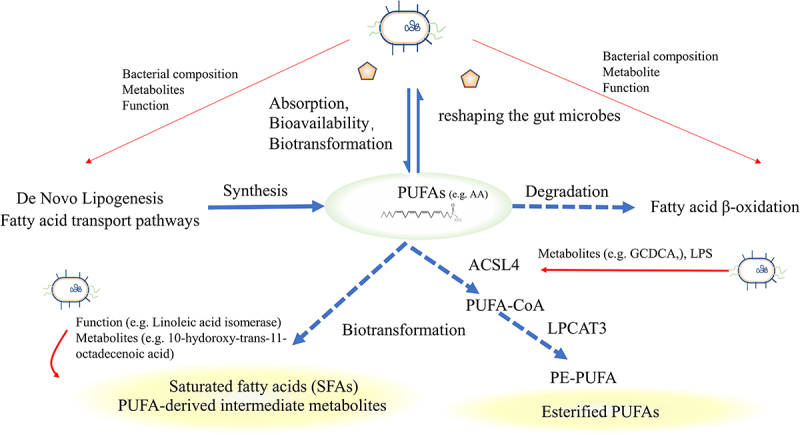
Fatty acid metabolism and intestinal microbiota. Exogenous PUFAs increase sensitivity to ferroptosis and shape the gut microbiota. The gut microbiota influences the balance of PUFAs by regulating lipid synthesis, degradation, and biotransformation. Microbiota can influence the expression of ACSL4, disturbing the esterification of PUFAs or facilitating the transformation of saturated fatty acids or PUFAs-derived intermediate metabolites, thereby influencing sensitivity to oxidation. PUFAs, Short-chain fatty acids; ACSL4, acyl-CoA synthetase long-chain family 4.

In the framework of ferroptosis, esterified PUFAs, rather than free PUFAs, are mainly affected by lipid peroxidation.^113^ Acyl-CoA synthetase long-chain family 4 (ACSL4), plays a crucial role in regulating ferroptosis by selectively converting long-chain PUFAs into their acyl-CoA esters, resulting in the formation of pro-ferroptotic lipid peroxidation products.^113^ ACSL4 catalyzes the activation of lysophosphatidylcholine acyltransferase 3 (LPCAT3), which then inserts acyl groups into lysophosphatidylethanolamine (LPE) to form PE-PUFAs that are directly oxidized by oxygenases.^98^ Thus, ACSL4 and LPCAT3 are essential factors that determine the sensitivity to ferroptosis.^114^ Increasing researches suggest that microbiota affects the esterification process to influence the ferroptosis. The metabolite glycochenodeoxycholate (GCDCA) produced by gut microbiota activates TfR-ACSL4-mediated ferroptosis^38^ and increases fecal lipid content while decreasing intestinal lipid digestibility.^115^ Lipopolysaccharides (LPS), a structural component of the microbiota, up-regulate the expression of ACSL4 to modulate ferroptosis in esophageal tissue by activating special protein 1 (Sp1).^52^ Bacteroides negatively regulates long-chain-fatty-acid-CoA ligase to regulate lipid metabolism and ferroptosis.^116^(Figure 4).

### Lipid peroxidation

The destruction of lipids caused by ROS, known as lipid peroxidation, is widely regarded as the primary biochemical event that induces ferroptosis and compromises the stability of cellular and organelle membranes. Carbon-carbon double bonds, particularly those found in PUFAs, are susceptible to oxidation by various oxidants from multiple sources (e.g., mitochondria electron transport chain, NOXs, Fenton reaction).^98^ However, some reports indicate that lipid autoxidation, rather than external oxidants, is the primary driver of ferroptosis, and iron promotes lipid autoxidation via the Fenton reaction, independence of any enzymatic process.^117^

Besides excessive production of ROS, disruption of antioxidant systems is also a major reason.

① The cyst(e)ine/GSH/GPX4 axis is the most well-known antioxidant system, and it has been widely recognized as the cornerstone in controlling ferroptosis.^7,118^ GSH catalyzes the reduction of lipid peroxidation to reduce oxidative stress and preserves the homeostasis of the cellular interior. Cystine is the substrate for GSH synthesis, whose uptake is controlled by cystine/glutamate transporter. When solute carrier family 7 member 11 (SLC7A11), a crucial component of the cystine/glutamate transporter on the cell membrane, is suppressed, it leads to the depletion of cystine and the buildup of lipid peroxide.^119^ GPX4, a critical regulator and marker of ferroptosis, utilizes GSH to convert toxic lipid hydroperoxides of PUFA to nontoxic lipid alcohols. Suppressing GPX4 results in decreased GSH synthesis and a rise in the buildup of lipid peroxidation, ultimately worsening ferroptosis.^8^ In addition, P53 represses SLC7A11, thereby inhibiting cystine uptake and rendering cells more susceptible to ferroptosis.^120^ A recent study demonstrates that capsiate, a metabolite produced by gut microbiota, activates Transient Receptor Potential Cation Channel Subfamily V Member 1 (TRPV1) to boost GPX4 expression and mitigates ferroptosis induced by intestinal IRI.^61^ Furthermore, research has demonstrated that a disruption of the intestinal microbiota caused by a high-fat diet leads to excessive activation of atrial ferroptosis, with a decrease of protein GPX4 and an increase of prostaglandin-endoperoxide synthase 2 (PTGS2).^121^ Butyrate inhibits the cystine/glutamate transporter system, inducing ferroptosis through various upstream molecular pathways, including RNA-binding motif protein 3 (RBM3), the Free fatty acid receptor 2 (FFAR2)-AKT-NRF2 axis, the FFAR2-mammalian target of rapamycin complex 1 (mTORC1) axis, or c-Fos.^56–59^ These findings demonstrate that butyrate contributes to GPX4-dependent ferroptosis. Neurotransmitters such as γ-aminobutyric acid (GABA), dopamine (DA), norepinephrine (NE), serotonin (5-HT), and histamine, which are produced by microorganisms, generally act on the nervous system through the gut-brain axis. 5-HT and 3-HA eliminate radicals to resist ferroptosis.^63^ Histamine deficiency disrupts histamine signaling, repressing the activation of signal transducer and activator of transcription 3 (STAT3), accompanied by decreased expression of SLC7A11, and accelerates myocardial ferroptosis.^64^ However, higher histamine levels related to gut microbiota dysbiosis can trigger ferroptosis in Perfluorooctanoic acid-induced abnormal embryonic development.^122^

② The Ferroptosis suppressor protein 1 (FSP1)- ubiquinone (CoQ) 10 axis operates in parallel with GPX4 to effectively inhibit ferroptosis.^123^ FSP1, which is also referred to as Apoptosis Inducing Factor Mitochondria Associated 2 (AIFM2), is capable of reducing CoQ to ubiquinol (CoQH2) on the plasma membrane and inner mitochondrial membrane, respectively. CoQH2 functions as a scavenging antioxidant that deactivates lipid peroxyl radicals, effectively inhibiting ferroptosis.^123,124^ Moreover, CoQ10 is the product of the mevalonate pathway and blocking this pathway hinders the highly effective synthesis of GPX4, making cells more vulnerable to ferroptosis.^125^ Existing evidence suggests that *Bacillus Calmette-Guérin* infection induces ferroptosis by decreasing the anti-ferroptosis regulators GPX4 and FSP1.^126^ Probiotic Strain *L. lactis MG1363-pMG36e-GLP-1* exerts neurotrophic effects via activating the Kelch-like ECH associated protein 1 (Keap1)/NRF2/GPX4 pathway to down-regulate ACSL4 and up-regulate FSP1 to suppress ferroptosis.^127^

③ The GTP cyclohydrolase-1 (GCH1)-tetrahydrobiopterin (BH4) axis, which operates in parallel with the GPX4 and FSP1 redox systems, plays a crucial role in preventing lipid peroxidation damage during ferroptosis induction in cancer cells. BH4, synthesized by GCH1, acts as a potent scavenger of free radicals. It safeguards lipid membranes from autoxidation by preventing reductions in the levels of phospholipids containing two polyunsaturated fatty acyl tails. Moreover, BH4 works in conjunction with vitamin E to enhance this protective effect.^128,129^ The research showed that by regulating the endogenous production of CoQ10, GCH1 and BH4/BH2 facilitated insensitivity to ferroptosis.^128^ Polybacterial infection inhibited the level BH4 in the mesenteric artery and colon, worsening atherosclerotic vascular disease.^130^ However, *Escherichia coli K1* increased GCH1^131^ or BH4^132^ expression in human brain microvascular endothelial cells and macrophage to evade surveillance by the immune system.

④ The thioredoxin system plays a vital role in sustaining cellular redox balance and enabling cancer cells to escape ferroptosis.^133^ Comprised of NADPH, thioredoxin reductase (TrxR), and thioredoxin (Trx), this system compensates for the absence of GSH biosynthesis in keratinocytes by the collaborative action of the SLC7A11 and the thioredoxin system.^134^ In this process, the transfer of electrons from NADPH to oxidized Trx by TrxR results in the conversion of oxidized Trx to its reduced dithiol form. The reduced Trx then undergoes an exchange of thiol and disulfide bonds in a reversible manner with several downstream proteins, such as antioxidant enzymes and apoptosis-regulating proteins.^135^ *Bifidobacterium animalis A12* and *Lactobacillus salivarius M18–6* activated thioredoxin system to alleviate alcohol injury in mice.^136^ Selenium-enriched probiotics increased blood glutathione peroxidase activity and tissue TrxR mRNA expression to keep pigs healthy under high ambient temperature.^137^

⑤ It is noteworthy that the susceptibility to ferroptosis is influenced by the enrichment of inducible nitric oxide synthase (iNOS)/NO• in activated M1 macrophages/microglia, but not in alternatively activated M2 macrophages/microglia.^138^ iNOS is responsible for producing nitric oxide (NO), which can automatically combine with superoxide to create peroxynitrite, a powerful trigger of oxidative stress. Despite not categorized as a member of the NOX family, iNOS is an important RNS-producing enzyme in the intestine.^139^ NO• produced by iNOS has the ability to engage with lipid intermediates produced by lipoxygenase (LOX). This indicates that the iNOS/NO• system serves as a robust regulator of ferroptotic death, resulting in the development of resistance of M1 form macrophages and microglia to ferroptosis triggers.^140^

**Figure 5.**
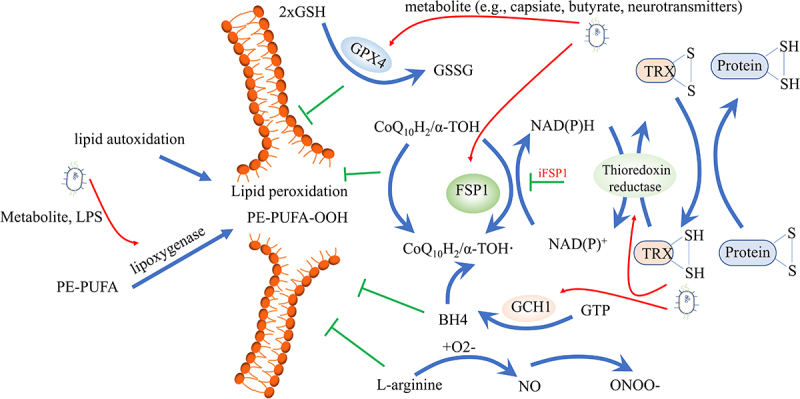
Intestinal microbiota affects lipid peroxidation. Different antioxidant systems are effective in preventing lipid peroxidation, including the cysteine/GSH/GPX4 axis, the FSP1-CoQ10 axis, the GCH1-BH4 axis, the thioredoxin system, and the iNOS/NO• system. On the other hand, lipid autoxidation and lipoxygenase can pose dangerous risk factors for inducing lipid peroxidation. Gut microbes can regulate both the antioxidant systems and lipoxygenase, influencing ferroptosis. GSH, glutathione; GPX4, glutathione peroxidase 4; FSP1, ferroptosis suppressor protein 1; CoQ10, Ubiquinone; GCH1, GTP cyclohydrolase-1; BH4, Tetrahydrobiopterin; iNOS, inducible nitric oxide synthase.

Lipids are oxidized through a direct pathway by a specific class of iron-containing enzymes known as oxygenases, such as LOX and NADPH-cytochrome P450 reductase (POR). This results in the initial accumulation of phospholipid hydroperoxides (PLOOH) and is believed to be involved in iron’s facilitation of the ferroptotic process.^114,141^ POR, in conjunction with NADH-cytochrome b5 reductase (CYB5R1), facilitates the transfer of electrons from NAD(P)H to oxygen, resulting in the generation of H_2_O_2_, which reacts with iron, leading to the formation of ROS that initiate the peroxidation of membrane phospholipids, ultimately causing the disruption of membrane integrity during the ferroptosis process.^142,143^ LOX enzymes, including 6 subtypes in humans (i.e., 5-LOX, p12-LOX, and 15-LOX-1), oxygenate PUFAs at various carbon positions, generating lipid products with significant biological activity. This contributes to the initiation of the ferroptosis signal. The initiation of the ferroptotic death signal occurs at the cellular membrane and is exacerbated by the lack of GPX4.^144^ Evidence suggests that diet-related microbial dysbiosis promotes 5-LOX mediated systemic neuroinflammation;^145^ LPS downregulates Lox5 messenger RNA expression;^146^ kombucha, a fermented tea containing a variety of acetic acid bacteria genera, yeasts, and a smaller proportion of lactic acid bacteria is against the 15-LOX.^147^ (Figure 5). Additionally, *Pseudomonas aeruginosa* utilizes its 15-LOX to trigger ferroptotic death in epithelial cells by oxidizing the host arachidonoyl-phosphatidylethanolamine into pro-ferroptotic 15-hydroperoxy- arachidonyl-PE (15-HpETE-PE).^148^ Based on this evidence, we speculate that the intestinal microbiota affects ferroptosis by interfering with iron-containing enzymes.

## Microbial regulation of ferroptosis in intestinal diseases

The intestinal microbiota plays a pivotal role in monitoring the occurrence of ferroptosis in intestinal diseases, more so than in diseases affecting other organs since the intestine is the first site of contact with dietary iron and the location where microorganisms colonize.^73^ The excessive oral administration of soluble iron has been associated with adverse alterations in gut microbiota in gastrointestinal diseases and an increase in the proliferation and attachment of enteric pathogens.^73,76^ A focus on ferroptosis in enterocytes or immunocytes and the role of microbiota in this process may provide a novel approach to the therapy of intestinal diseases. Since different diseases may share common pathogenic mechanisms, research on intestinal diseases can provide valuable insights for treating diseases in other systems. For instance, in osteoarthritis, IRI, and ultraviolet B-induced skin inflammation, gut microbiota and its metabolite, capsiate, have been found to inhibit the expression of HIF-1a and metastasis-associated lung adenocarcinoma transcript 1 (MALAT1), intracellular ROS, and reduce ferroptosis.^149,150^ The AKT/Glycogen synthase kinase 3β (GSK3β)/NRF2 pathway or the Adenosine 5‘-monophosphate (AMP)-activated protein kinase (AMPK)/NRF2 pathways were activated to alleviate diseases by daidzein liberated by *Limosilactobacillus vaginalis* β-galactosidase in acetaminophen-induced hepatotoxicity,^151^ by sulforaphane in diabetic cardiomyopathy,^152^ and by roxadustat (FG-4592) in folic acid-induced kidney injury.^153^

### Colorectal cancer (CRC)

CRC is the third most prevalent malignant cancer worldwide, with approximately 1.2 million new cases and 600,000 deaths annually.^154^ For the pathogenesis of CRC, besides the alteration in genes, dietary habits and gut microbes are critical in the pathogenesis. Dysbiosis promotes bacterial translocation, generates ROS/RNS, causes DNA damage and oxidative stress, activates macrophage to release TNF-α, leading to chromosome instability and cell transformation, all of which result in aberrant proliferation and CRC formation.^155^ For changes that occur before cancer, inhibiting ROS using ferroptosis inhibitors may be a feasible method. However, once the tumor forms or exhibits drug resistance, the effective treatment involves activating ferroptosis by consuming GSH, stimulating ROS, and increasing iron concentration.^7,156,157^ *N*-3 PUFAs increase the sensitivity of CRC cells to ferroptosis-mediated cell death and modulate the gut microbiome, increasing the presence of *Lactobacillus* and *Bifidobacteria*. These changes have prebiotic effects, reducing inflammation.^56^ Consumption of *n*-3 PUFA and butyrate potentially reduce colon tumor formation by promoting a Gpx4-dependent, lipid oxidation-sensitive and mitochondrial dependent cell death pathway in the colonic mucosa.^56^

### Ischemia-reperfusion injury (IRI)

Intestinal IRI is a potentially fatal condition caused by the abrupt decrease in blood flow to the intestines, followed by reoxygenation after blood supply is reinstated. This leads to acute intestinal barrier disruption and bacterial translocation.^158,159^ ROS generation, decreased GSH levels and superoxide dismutase activity, along with lipid peroxidation are observed in intestinal IRI and execution of ferroptosis.^160,161^ The evidence showed that inhibition of ferroptosis by Liproxstatin-1, Deferoxamine or the ACSL4 inhibition ameliorated IRI.^158,162^ In addition, capsiate, derived from the gut microbiota, activates TRPV1 to enhance GPX4 expression, thereby mitigating the deleterious effects of ferroptosis on intestinal IRI.^61^

### IBD

IBD represents a collection of chronic and recurrent inflammatory conditions that affect the gastrointestinal tract, including Crohn’s disease (CD) and ulcerative colitis (UC).^163^ Although the etiology of IBD remains elusive, accumulating evidence indicates a pivotal role of the gut microbiota in the pathogenesis of intestinal inflammation. Remarkably, the role of ferroptosis in the pathogenesis and progression of IBD has been reported in both human and murine studies.^164^ Inflamed areas within the colonic mucosa are characterized by high levels of H_2_O_2_ resulting from the immune cell-initiated release of oxygen and high concentrations of Fe^2+^ from degraded heme molecules. These conditions create a favorable environment for ferroptosis.^165^ Specifically, inhibitors of ferroptosis such as Ferrostatin-1, Liproxstatin-1, and Deferoxamine, or inhibiting LOX enzymes, have been demonstrated to alleviate the symptoms of colitis induced by DSS in mice,^156,166^ suggesting a potential therapeutic strategy for IBD. Microbial disorders, which involve the reduction of bacteria producing SCFAs, are considered to be related to IBD because they increase vulnerability to inflammation in IBD by modifying the infiltration of immunocytes.^167^ Furthermore, one study showed gut microbiota controlled susceptibility to colitis and ferroptosis early in life via microbial-derived ether lipids.^168^ Probiotics maintain gut homeostasis by strengthening the gut barrier function, checking the ROS generation, maintaining the antioxidant level, and modulating the immunity.^169,170^ *Escherichia coli* was considered as an iron scavenger, chelating free iron to inhibit hydroxyl radical formation at inflammatory sites in patients with UC.^165^ Furthermore, treatment with OTSSP167, a maternal embryonic leucine zipper kinase (MELK)-selective inhibitor, was shown to improve gut microbial composition, decrease ferroptosis in intestinal epithelial cells, and reduce the infiltration and polarization of macrophages in a model of colitis and colitis-associated carcinogenesis.^4^ Modified probiotics have a more potent effect in reducing ROS. For example, *Escherichia coli Nissle 1917*, an oral probiotic genetically engineered to overexpress catalase and superoxide dismutase, effectively alleviated inflammation, repaired epithelial barriers in the colon, and improved microbial communities.^171^

### Other intestinal diseases

In addition to the three diseases mentioned above, intestinal damage can also occur due to multiple factors related to ferroptosis and gut microbiota. *Pseudomonas aeruginosa* infection in intestines led to ferroptosis induced by radiation exposure, achieved by suppressing the host’s anti-ferroptotic system, and employing bacterial 15-LOX to generate 15-HpETE-PE.^148,172^ Intestinal ferroptosis mediated by Bacteroidaceae occurred due to benzene exposure, but it was significantly reversed by oral probiotics.^73^ Additionally, a mixture of *Lactobacillus* spp. in murine models effectively reduced irinotecan-induced diarrhea by decreasing β-glucuronidase expression and ROS levels, while protecting the gut epithelium from microbial dysbiosis and proliferative crypt injury.^173^

## Conclusion

Iron is ubiquitous in our daily lives and has a close and intricate association with the physiological functions of the host and intestinal microbiota. Ferroptosis is considered to be a major contributor to tissue damage resulting from cell rupture, which in turn triggers necroinflammatory processes.^174^ Numerous genes and signaling pathways have been shown to be implicated in ferroptosis, constituting a complex regulatory system such that modulation of a single gene may not significantly affect ferroptotic processes. The effectiveness of ferroptosis inhibitors such as Ferrostatin-1, and inducers such as RSL3 has been confirmed in animal models of multiple diseases. Furthermore, Chelating drugs and their metal complexes are currently widely used in clinical practice, primarily for the treatment of transfusional iron overload,^175,176^ and in pilot clinical trials for treating amyotrophic lateral sclerosis.^177^ Inhibitors that regulate iron metabolism or function as antioxidants in targeting neurodegeneration have been widely applied in clinical trials.^178,179^ In the future, reagents that target ferroptosis show promise for clinical applications in treating a wider range of diseases. Former scientific inquiries have demonstrated significant alterations in the gut microbial community of patients suffering from diverse medical conditions. The metabolic byproducts produced by the gut microbiota modulate various physiological processes, such as signaling pathways and immune system responses. Additionally, these byproducts exhibit antibiotic activity, regulate ferroptosis and shape the colon’s environment. Despite the significant impact of gut microbiota on the regulation of various physiological processes, there is a paucity of studies investigating their involvement in ferroptosis. Furthermore, the precise microbes and small molecules that modulate ferroptosis and the temporal window during which they exert their effects remain elusive. Presently, research primarily focuses on predicting interactions between representative microbial genera and genes associated with ferroptosis or conducting correlation analyses between them. In other words, the evidence of the relationship between microbe and ferroptosis is indirect and insufficient. Moreover, the available studies rarely explore the mechanisms by which bacteria affect ferroptosis. The aforementioned hypothesis possesses the potential to elucidate the intricate relationship between gut microbiota and ferroptosis, thereby hastening pertinent research endeavors. In the future, regulating intestinal microbiota to promote or reduce ferroptosis may become a promising approach for treating corresponding diseases.

## References

[1] Zheng J, Conrad M. The metabolic underpinnings of ferroptosis. Cell Metab. 2020;32(6):920–22. doi:10.1016/j.cmet.2020.10.011. 33217331

[2] Bai Y, Gao F, Li D, Ji S, Zhang S, Guo W, Li B. The effect of FOXP3+ regulatory T cells on Infectious and inflammatory diseases. Infect Microbes Dis. 2021;3(4):187–197. doi:10.1097/IM9.0000000000000070.

[3] Li X, Wang TX, Huang X, Li Y, Sun T, Zang S, Guan KL, Xiong Y, Liu J, Yuan H-X, et al. Targeting ferroptosis alleviates methionine-choline deficient (MCD)-diet induced NASH by suppressing liver lipotoxicity. Liver Int. 2020;40(6):1378–1394. doi:10.1111/liv.14428. 32145145

[4] Tang B, Zhu J, Fang S, Wang Y, Vinothkumar R, Li M, Weng Q, Zheng L, Yang Y, Qiu R, et al. Pharmacological inhibition of MELK restricts ferroptosis and the inflammatory response in colitis and colitis-propelled carcinogenesis. Free Radic Biol Med. 2021;172:312–329. doi:10.1016/j.freeradbiomed.2021.06.012. 34144192

[5] Wu JR, Tuo QZ, Lei P. Ferroptosis, a recent defined form of critical cell death in neurological disorders. J Mol Neurosci. 2018;66(2):197–206. doi:10.1007/s12031-018-1155-6. 30145632

[6] Li S, Wang Q, Su B. mTOR-Mediated cell death and infection. Infect Microbes Dis. 2021;3(2):57–68. doi:10.1097/IM9.0000000000000063.

[7] Zhao L, Zhou X, Xie F, Zhang L, Yan H, Huang J, Zhang C, Zhou F, Chen J, Zhang L, et al. Ferroptosis in cancer and cancer immunotherapy. Cancer Commun (Lond). 2022;42(2):88–116. doi:10.1002/cac2.12250. 35133083 PMC8822596

[8] Dixon SJ, Lemberg KM, Lamprecht MR, Skouta R, Zaitsev EM, Gleason CE, Patel DN, Bauer A, Cantley A, Yang W, et al. Ferroptosis: an iron-dependent form of nonapoptotic cell death. Cell. 2012;149(5):1060–1072. doi:10.1016/j.cell.2012.03.042. 22632970 PMC3367386

[9] Chen X, Kang R, Kroemer G, Tang D. Ferroptosis in infection, inflammation, and immunity. J Exp Med. 2021;218(6):e20210518. doi:10.1084/jem.20210518. 33978684 PMC8126980

[10] Lee YS, Lee DH, Choudry HA, Bartlett DL, Lee YJ. Ferroptosis-induced endoplasmic reticulum stress: cross-talk between ferroptosis and apoptosis. Mol Cancer Res. 2018;16(7):1073–1076. doi:10.1158/1541-7786.MCR-18-0055. 29592897 PMC6030493

[11] Zhou B, Liu J, Kang R, Klionsky DJ, Kroemer G, Tang D. Ferroptosis is a type of autophagy-dependent cell death. Semin Cancer Biol. 2020;66:89–100. doi:10.1016/j.semcancer.2019.03.002. 30880243

[12] Yu H, Guo P, Xie X, Wang Y, Chen G. Ferroptosis, a new form of cell death, and its relationships with tumourous diseases. J Cell Mol Med. 2017;21(4):648–657. doi:10.1111/jcmm.13008. 27860262 PMC5345622

[13] Caruso R, Lo BC, Núñez G. Host-microbiota interactions in inflammatory bowel disease. Nat Rev Immunol. 2020;20(7):411–426. doi:10.1038/s41577-019-0268-7. 32005980

[14] Tang WHW, Li DY, Hazen SL. Dietary metabolism, the gut microbiome, and heart failure. Nat Rev Cardiol. 2019;16(3):137–154. doi:10.1038/s41569-018-0108-7. 30410105 PMC6377322

[15] Neish AS, Jones RM. Redox signaling mediates symbiosis between the gut microbiota and the intestine. Gut Microbes. 2014;5(2):250–253. doi:10.4161/gmic.27917. 24637602 PMC4063853

[16] Kotloff KL, Riddle MS, Platts-Mills JA, Pavlinac P, Zaidi AKM. Shigellosis. Lancet. 2018;391(10122):801–812. doi:10.1016/S0140-6736(17)33296-8. 29254859

[17] Tang WHW, Bäckhed F, Landmesser U, Hazen SL. Intestinal microbiota in cardiovascular health and disease: JACC state-of-the-art review. J Am Coll Cardiol. 2019;73(16):2089–2105. doi:10.1016/j.jacc.2019.03.024. 31023434 PMC6518422

[18] Larsson E, Tremaroli V, Lee YS, Koren O, Nookaew I, Fricker A, Nielsen J, Ley RE, Bäckhed F. Analysis of gut microbial regulation of host gene expression along the length of the gut and regulation of gut microbial ecology through MyD88. Gut. 2012;61(8):1124–1131. doi:10.1136/gutjnl-2011-301104. 22115825 PMC3388726

[19] Peng M, Lee SH, Rahaman SO, Biswas D. Dietary probiotic and metabolites improve intestinal homeostasis and prevent colorectal cancer. Food Funct. 2020;11(12):10724–10735. doi:10.1039/D0FO02652B. 33231228

[20] Levy M, Kolodziejczyk AA, Thaiss CA, Elinav E. Dysbiosis and the immune system. Nat Rev Immunol. 2017;17(4):219–232. doi:10.1038/nri.2017.7. 28260787

[21] Gershon MD, Margolis KG. The gut, its microbiome, and the brain: connections and communications. J Clin Invest. 2021;131(18). doi:10.1172/JCI143768. PMC8439601 34523615

[22] Ahlawat S, Asha A, Sharma KK. Gut-organ axis: a microbial outreach and networking. Lett Appl Microbiol. 2021;72(6):636–668. doi:10.1111/lam.13333. 32472555

[23] Shanmugam NK, Trebicka E, Fu LL, Shi HN, Cherayil BJ. Intestinal inflammation modulates expression of the iron-regulating hormone hepcidin depending on erythropoietic activity and the commensal microbiota. J Immunol. 2014;193(3):1398–1407. doi:10.4049/jimmunol.1400278. 24973448 PMC4108560

[24] Hu C, Liu M, Tang L, Liu H, Sun B, Chen L. Probiotic intervention mitigates the metabolic disturbances of perfluorobutanesulfonate along the gut-liver axis of zebrafish. Chemosphere. 2021;284(131374):131374. doi:10.1016/j.chemosphere.2021.131374. 34217933

[25] Zhang L, Kang H, Zhang W, Wang J, Liu Z, Jing J, Han L, Gao A. Probiotics ameliorate benzene-induced systemic inflammation and hematopoietic toxicity by inhibiting Bacteroidaceae-mediated ferroptosis. Sci Total Environ. 2023;899(165678):165678. doi:10.1016/j.scitotenv.2023.165678. 37478946

[26] Grzeszczak K, Kwiatkowski S, Kosik-Bogacka D. The role of Fe, zn, and Cu in pregnancy. Biomolecules. 2020;10(8):1176. doi:10.3390/biom10081176. 32806787 PMC7463674

[27] Dev S, Babitt JL. Overview of iron metabolism in health and disease. Hemodial Int. 2017;21 Suppl 1(Suppl 1):S6–s20. doi:10.1111/hdi.12542. 28296010 PMC5977983

[28] Chen X, Yu C, Kang R, Kroemer G, Tang D. Cellular degradation systems in ferroptosis. Cell Death Differ. 2021;28(4):1135–1148. doi:10.1038/s41418-020-00728-1. 33462411 PMC8027807

[29] Vogt AS, Arsiwala T, Mohsen M, Vogel M, Manolova V, Bachmann MF. On iron metabolism and its regulation. Int J Mol Sci. 2021;22(9):4591. doi:10.3390/ijms22094591. 33925597 PMC8123811

[30] Lane DJ, Merlot AM, Huang ML, Bae DH, Jansson PJ, Sahni S, Kalinowski DS, Richardson DR. Cellular iron uptake, trafficking and metabolism: key molecules and mechanisms and their roles in disease. Biochim Biophys Acta. 2015;1853(5):1130–1144. doi:10.1016/j.bbamcr.2015.01.021. 25661197

[31] Sharp PA. Intestinal iron absorption: regulation by dietary & systemic factors. Int J Vitamin Nutr Res. 2010;80(45):231–242. doi:10.1024/0300-9831/a000029. 21462105

[32] Anderson GJ, Vulpe CD. Mammalian iron transport. Cell Mol Life Sci. 2009;66(20):3241–3261. doi:10.1007/s00018-009-0051-1. 19484405 PMC11115736

[33] Viña J, Tarazona-Santabalbina FJ, Pérez-Ros P, Martínez-Arnau FM, Borras C, Olaso-Gonzalez G, Salvador-Pascual A, Gomez-Cabrera MC. Biology of frailty: modulation of ageing genes and its importance to prevent age-associated loss of function. Mol Aspects Med. 2016;50:88–108. doi:10.1016/j.mam.2016.04.005. 27164416

[34] Kawabata H. Transferrin and transferrin receptors update. Free Radic Biol Med. 2019;133:46–54. doi:10.1016/j.freeradbiomed.2018.06.037. 29969719

[35] Brown CW, Amante JJ, Chhoy P, Elaimy AL, Liu H, Zhu LJ, Baer CE, Dixon SJ, Mercurio AM. Prominin2 drives ferroptosis resistance by stimulating iron export. Dev Cell. 2019;51(5):575–86.e4. doi:10.1016/j.devcel.2019.10.007. 31735663 PMC8316835

[36] Harrison PM, Arosio P. The ferritins: molecular properties, iron storage function and cellular regulation. Biochim Biophys Acta. 1996;1275(3):161–203. doi:10.1016/0005-2728(96)00022-9. 8695634

[37] Schöttker B, Gào X, Jansen EH, Brenner H. Associations of human colorectal adenoma with serum biomarkers of body iron stores, inflammation and antioxidant protein thiols. Antioxidants (Basel). 2021;10(8):1195. doi:10.3390/antiox10081195. 34439443 PMC8388983

[38] Liu S, Gao Z, He W, Wu Y, Liu J, Zhang S, Yan L, Mao S, Shi X, Fan W, et al. The gut microbiota metabolite glycochenodeoxycholate activates TFR-ACSL4-mediated ferroptosis to promote the development of environmental toxin–linked MAFLD. Free Radic Biol Med. 2022;193(Pt 1):213–226. doi:10.1016/j.freeradbiomed.2022.10.270. 36265794

[39] Vanoaica L, Darshan D, Richman L, Schümann K, Kühn LC. Intestinal ferritin H is required for an accurate control of iron absorption. Cell Metab. 2010;12(3):273–282. doi:10.1016/j.cmet.2010.08.003. 20816093

[40] Torti FM, Torti SV. Regulation of ferritin genes and protein. Blood. 2002;99(10):3505–3516. doi:10.1182/blood.V99.10.3505. 11986201

[41] Hider RC, Kong XL. Glutathione: a key component of the cytoplasmic labile iron pool. Biometals. 2011;24(6):1179–1187. doi:10.1007/s10534-011-9476-8. 21769609

[42] Mancias JD, Wang X, Gygi SP, Harper JW, Kimmelman AC. Quantitative proteomics identifies NCOA4 as the cargo receptor mediating ferritinophagy. Nature. 2014;509(7498):105–109. doi:10.1038/nature13148. 24695223 PMC4180099

[43] Kernan KF, Carcillo JA. Hyperferritinemia and inflammation. Int Immunol. 2017;29(9):401–409. doi:10.1093/intimm/dxx031. 28541437 PMC5890889

[44] Gammella E, Buratti P, Cairo G, Recalcati S. The transferrin receptor: the cellular iron gate. Metallomics. 2017;9(10):1367–1375. doi:10.1039/C7MT00143F. 28671201

[45] Lambert LA, Mitchell SL. Molecular evolution of the transferrin receptor/glutamate carboxypeptidase II family. J Mol Evol. 2007;64(1):113–128. doi:10.1007/s00239-006-0137-4. 17160644

[46] Seyoum Y, Baye K, Humblot C. Iron homeostasis in host and gut bacteria - a complex interrelationship. Gut Microbes. 2021;13(1):1–19. doi:10.1080/19490976.2021.1874855. PMC7872071 33541211

[47] Anderson GJ, Frazer DM. Hepatic iron metabolism. Semin Liver Dis. 2005;25(4):420–432. doi:10.1055/s-2005-923314. 16315136

[48] Qiao B, Sugianto P, Fung E, Del-Castillo-Rueda A, Moran-Jimenez MJ, Ganz T, Nemeth E. Hepcidin-induced endocytosis of ferroportin is dependent on ferroportin ubiquitination. Cell Metab. 2012;15(6):918–924. doi:10.1016/j.cmet.2012.03.018. 22682227 PMC3372862

[49] Daher R, Karim Z. Iron metabolism: state of the art. Transfus Clin Biol. 2017;24(3):115–119. doi:10.1016/j.tracli.2017.06.015. 28694024

[50] Wang Y, Wu Y, Wang Y, Xu H, Mei X, Yu D, Wang Y, Li W. Antioxidant properties of probiotic bacteria. Nutrients. 2017;9(5):521. doi:10.3390/nu9050521. 28534820 PMC5452251

[51] Asnicar F, Berry SE, Valdes AM, Nguyen LH, Piccinno G, Drew DA, Leeming E, Gibson R, Le Roy C, Khatib HA, et al. Microbiome connections with host metabolism and habitual diet from 1,098 deeply phenotyped individuals. Nat Med. 2021;27(2):321–332. doi:10.1038/s41591-020-01183-8. 33432175 PMC8353542

[52] Liu S, Tang Y, Liu L, Yang L, Li P, Liu X, Yin H. Proteomic analysis reveals that ACSL4 activation during reflux esophagitis contributes to ferroptosis-mediated esophageal mucosal damage. Eur J Pharmacol. 2022;931(175175):175175. doi:10.1016/j.ejphar.2022.175175. 35921957

[53] Miao Z, Miao Z, Teng X, Xu S. Melatonin alleviates lead-induced fatty liver in the common carps (Cyprinus carpio) via gut-liver axis. Environ Pollut. 2023;317(120730):120730. doi:10.1016/j.envpol.2022.120730. 36427828

[54] Rose S, Bennuri SC, Davis JE, Wynne R, Slattery JC, Tippett M, Delhey L, Melnyk S, Kahler SG, MacFabe DF, et al. Butyrate enhances mitochondrial function during oxidative stress in cell lines from boys with autism. Transl Psychiatry. 2018;8(1):42. doi:10.1038/s41398-017-0089-z. 29391397 PMC5804031

[55] Li X, Dong L, Li A, Yi J, Brotto M, Zhou J. Butyrate ameliorates mitochondrial respiratory capacity of the motor-neuron-like cell line NSC34-G93A, a cellular model for ALS. Biomolecules. 2022;12(2):333. doi:10.3390/biom12020333. 35204833 PMC8869540

[56] Chapkin RS, Navarro SL, Hullar MAJ, Lampe JW. Diet and gut Microbes act coordinately to enhance programmed cell death and reduce colorectal cancer risk. Dig Dis Sci. 2020;65(3):840–851. doi:10.1007/s10620-020-06106-8. 32006211 PMC7605510

[57] Wang G, Qin S, Chen L, Geng H, Zheng Y, Xia C, Yao J, Deng L. Butyrate dictates ferroptosis sensitivity through FFAR2-mTOR signaling. Cell Death Disease. 2023;14(4):292. doi:10.1038/s41419-023-05778-0. 37185889 PMC10130170

[58] Wang Z, Shu W, Zhao R, Liu Y, Wang H. Sodium butyrate induces ferroptosis in endometrial cancer cells via the RBM3/SLC7A11 axis. Apoptosis. 2023;28(7–8):1168–1183. doi:10.1007/s10495-023-01850-4. 37170022

[59] He Y, Ling Y, Zhang Z, Mertens RT, Cao Q, Xu X, Guo K, Shi Q, Zhang X, Huo L, et al. Butyrate reverses ferroptosis resistance in colorectal cancer by inducing c-Fos-dependent xCT suppression. Redox Biol. 2023;65(102822):102822. doi:10.1016/j.redox.2023.102822. 37494767 PMC10388208

[60] Das NK, Schwartz AJ, Barthel G, Inohara N, Liu Q, Sankar A, Hill DR, Ma X, Lamberg O, Schnizlein MK, et al. Microbial metabolite signaling is required for systemic iron homeostasis. Cell Metab. 2020;31(1):115–30.e6. doi:10.1016/j.cmet.2019.10.005. 31708445 PMC6949377

[61] Deng F, Zhao BC, Yang X, Lin ZB, Sun QS, Wang YF, Yan ZZ, Liu W-F, Li C, Hu J-J, et al. The gut microbiota metabolite capsiate promotes Gpx4 expression by activating TRPV1 to inhibit intestinal ischemia reperfusion-induced ferroptosis. Gut Microbes. 2021;13(1):1–21. doi:10.1080/19490976.2021.1902719. PMC8009132 33779497

[62] D’Amico D, Andreux PA, Valdés P, Singh A, Rinsch C, Auwerx J. Impact of the natural compound urolithin a on health, disease, and aging. Trends Mol Med. 2021;27(7):687–699. doi:10.1016/j.molmed.2021.04.009. 34030963

[63] Liu D, Liang CH, Huang B, Zhuang X, Cui W, Yang L, Yang Y, Zhang Y, Fu X, Zhang X, et al. Tryptophan metabolism acts as a new anti-ferroptotic pathway to mediate tumor growth. Adv Sci. 2023;10(6):e2204006. doi:10.1002/advs.202204006. PMC9951368 36627132

[64] Zhu X, Wang X, Zhu B, Ding S, Shi H, Yang X. Disruption of histamine/H(1)R-STAT3-SLC7A11 axis exacerbates doxorubicin-induced cardiac ferroptosis. Free Radic Biol Med. 2022;192:98–114. doi:10.1016/j.freeradbiomed.2022.09.012. 36165929

[65] Zhang Y, Zhang P, Li Y. Gut microbiota-mediated ferroptosis contributes to mercury exposure-induced brain injury in common carp. Metallomics. 2022;14(1):mfab072. doi:10.1093/mtomcs/mfab072. 34905050

[66] Dar HH, Tyurina YY, Mikulska-Ruminska K, Shrivastava I, Ting HC, Tyurin VA, Krieger J, St. Croix CM, Watkins S, Bayir E, et al. Pseudomonas aeruginosa utilizes host polyunsaturated phosphatidylethanolamines to trigger theft-ferroptosis in bronchial epithelium. J Clin Invest. 2018;128(10):4639–4653. doi:10.1172/JCI99490. 30198910 PMC6159971

[67] Amaral EP, Costa DL, Namasivayam S, Riteau N, Kamenyeva O, Mittereder L, Mayer-Barber KD, Andrade BB, Sher A. A major role for ferroptosis in Mycobacterium tuberculosis–induced cell death and tissue necrosis. J Exp Med. 2019;216(3):556–570. doi:10.1084/jem.20181776. 30787033 PMC6400546

[68] Yao C, Lan D, Li X, Wang Y, Qi S, Liu Y. Porphyromonas gingivalis is a risk factor for the development of nonalcoholic fatty liver disease via ferroptosis. Microbes Infect. 2023;25(1–2):105040. doi:10.1016/j.micinf.2022.105040. 35987459

[69] Furumoto H, Nanthirudjanar T, Kume T, Izumi Y, Park SB, Kitamura N, Kishino S, Ogawa J, Hirata T, Sugawara T. 10-oxo-trans-11-octadecenoic acid generated from linoleic acid by a gut lactic acid bacterium Lactobacillus plantarum is cytoprotective against oxidative stress. Toxicol Appl Pharmacol. 2016;296:1–9. doi:10.1016/j.taap.2016.02.012. 26879219

[70] Yang M, Lu Z, Li F, Shi F, Zhan F, Zhao L, Li Y, Li J, Lin L, Qin Z. Escherichia coli induced ferroptosis in red blood cells of grass carp (Ctenopharyngodon idella). Fish Shellfish Immunol. 2021;112:159–167. doi:10.1016/j.fsi.2020.09.036. 33017637

[71] Wen Y, Wang Y, Chen S, Zhou X, Zhang Y, Yang D, Núñez G, Liu Q. Dysregulation of cytosolic c-di-GMP in Edwardsiella piscicida promotes cellular non-canonical ferroptosis. Front Cell Infect Microbiol. 2022;12:825824. doi:10.3389/fcimb.2022.825824. 35186798 PMC8855483

[72] Skrypnik K, Bogdański P, Sobieska M, Schmidt M, Suliburska J. Influence of multistrain probiotic and iron supplementation on iron status in rats. J Trace Elements Med Biol. 2021;68:126849. doi:10.1016/j.jtemb.2021.126849. 34488183

[73] Qi X, Zhang Y, Guo H, Hai Y, Luo Y, Yue T. Mechanism and intervention measures of iron side effects on the intestine. Crit Rev Food Sci Nutr. 2020;60(12):2113–2125. doi:10.1080/10408398.2019.1630599. 31232087

[74] Bessman NJ, Mathieu JRR, Renassia C, Zhou L, Fung TC, Fernandez KC, Austin C, Moeller JB, Zumerle S, Louis S, et al. Dendritic cell–derived hepcidin sequesters iron from the microbiota to promote mucosal healing. Sci. 2020;368(6487):186–189. doi:10.1126/science.aau6481. PMC7724573 32273468

[75] Stein J, Bager P, Befrits R, Gasche C, Gudehus M, Lerebours E, Magro F, Mearin F, Mitchell D, Oldenburg B, et al. Anaemia management in patients with inflammatory bowel disease: routine practice across nine European countries. Eur J Gastroenterol Hepatol. 2013;25(12):1456–1463. doi:10.1097/MEG.0b013e328365ca7f. 24100539

[76] Zhao N, Liu JM, Yang FE, Ji XM, Li CY, Lv SW, Wang S. A novel mediation strategy of DSS-Induced colitis in mice Based on an iron-enriched probiotic and in vivo bioluminescence tracing. J Agric Food Chem. 2020;68(43):12028–12038. doi:10.1021/acs.jafc.0c05260. 33052690

[77] Fikri B, Ridha NR, Putri SH, Salekede SB, Juliaty A, Tanjung C, Massi N. Effects of probiotics on immunity and iron homeostasis: a mini-review. Clin Nutr ESPEN. 2022;49:24–27. doi:10.1016/j.clnesp.2022.03.031. 35623819

[78] Saha M, Sarkar S, Sarkar B, Sharma BK, Bhattacharjee S, Tribedi P. Microbial siderophores and their potential applications: a review. Environ Sci Pollut Res Int. 2016;23(5):3984–3999. doi:10.1007/s11356-015-4294-0. 25758420

[79] Garénaux A, Caza M, Dozois CM. The ins and outs of siderophore mediated iron uptake by extra-intestinal pathogenic Escherichia coli. Vet Microbiol. 2011;153(1–2):89–98. doi:10.1016/j.vetmic.2011.05.023. 21680117

[80] Ren Z, Zhao Z, Wang Y, Huang K. Preparation of selenium/zinc-enriched probiotics and their effect on blood selenium and zinc concentrations, antioxidant capacities, and intestinal microflora in canine. Biol Trace Elem Res. 2011;141(1–3):170–183. doi:10.1007/s12011-010-8734-x. 20563665

[81] Gerner RR, Nuccio SP, Raffatellu M. Iron at the host-microbe interface. Mol Aspects Med. 2020;75(100895):100895. doi:10.1016/j.mam.2020.100895. 32883564 PMC7554189

[82] Tang Z, Ju Y, Dai X, Ni N, Liu Y, Zhang D, Gao H, Sun H, Zhang J, Gu P, et al. HO-1-mediated ferroptosis as a target for protection against retinal pigment epithelium degeneration. Redox Biol. 2021;43(101971):101971. doi:10.1016/j.redox.2021.101971. 33895485 PMC8099560

[83] Onyiah JC, Sheikh SZ, Maharshak N, Steinbach EC, Russo SM, Kobayashi T, Mackey LC, Hansen JJ, Moeser AJ, Rawls JF, et al. Carbon monoxide and heme oxygenase-1 prevent intestinal inflammation in mice by promoting bacterial clearance. Gastroenterology. 2013;144(4):789–798. doi:10.1053/j.gastro.2012.12.025. 23266559 PMC3608700

[84] Onyiah JC, Sheikh SZ, Maharshak N, Otterbein LE, Plevy SE. Heme oxygenase-1 and carbon monoxide regulate intestinal homeostasis and mucosal immune responses to the enteric microbiota. Gut Microbes. 2014;5(2):220–224. doi:10.4161/gmic.27290. 24637794 PMC4063848

[85] Zhang P, Han X, Zhang X, Zhu X. Lactobacillus acidophilus ATCC 4356 alleviates renal ischemia-reperfusion injury through antioxidant stress and anti-inflammatory responses and improves intestinal microbial distribution. Front Nutr. 2021;8(667695):667695. doi:10.3389/fnut.2021.667695. 34046422 PMC8144323

[86] Song X, Zhao Z, Zhao Y, Jin Q, Li S. Protective effects of Bacillus coagulans JA845 against D-Galactose/AlCl(3)-induced cognitive decline, oxidative stress and neuroinflammation. J Microbiol Biotechnol. 2022;32(2):212–219. doi:10.4014/jmb.2111.11031. 34954699 PMC9628844

[87] Xu C, Qiao L, Ma L, Guo Y, Dou X, Yan S, Zhang B, Román A. biogenic selenium nanoparticles synthesized by Lactobacillus casei ATCC 393 alleviate intestinal epithelial barrier dysfunction caused by oxidative stress via Nrf2 signaling-mediated mitochondrial pathway. Int J Nanomedicine. 2019;14:4491–4502. doi:10.2147/IJN.S199193. 31417254 PMC6593357

[88] El-Baz AM, El-Ganiny AM, Hellal D, Anwer HM, El-Aziz HAA, Tharwat IE, El-Adawy MA, Helal SEDM, Mohamed MTA, Azb TM, et al. Valuable effects of lactobacillus and citicoline on steatohepatitis: role of Nrf2/HO-1 and gut microbiota. AMB Express. 2023;13(1):57. doi:10.1186/s13568-023-01561-8. 37291355 PMC10250290

[89] Yang S, Ouyang J, Lu Y, Harypursat V, Chen Y. A dual role of heme oxygenase-1 in tuberculosis. Front Immunol. 2022;13:842858. doi:10.3389/fimmu.2022.842858. 35281042 PMC8913507

[90] Lloyd RV, Hanna PM, Mason RP. The origin of the hydroxyl radical oxygen in the Fenton reaction. Free Radic Biol Med. 1997;22(5):885–888. doi:10.1016/S0891-5849(96)00432-7. 9119257

[91] Pilarczyk-Zurek M, Strus M, Adamski P, Heczko PB. The dual role of Escherichia coli in the course of ulcerative colitis. BMC Gastroenterol. 2016;16(1):128. doi:10.1186/s12876-016-0540-2. 27724868 PMC5057264

[92] Keshavarzian A, Banan A, Farhadi A, Komanduri S, Mutlu E, Zhang Y, Fields JZ. Increases in free radicals and cytoskeletal protein oxidation and nitration in the colon of patients with inflammatory bowel disease. Gut. 2003;52(5):720–728. doi:10.1136/gut.52.5.720. 12692059 PMC1773652

[93] Gaschler MM, Hu F, Feng H, Linkermann A, Min W, Stockwell BR. Determination of the subcellular localization and mechanism of action of ferrostatins in suppressing ferroptosis. ACS Chem Biol. 2018;13(4):1013–1020. doi:10.1021/acschembio.8b00199. 29512999 PMC5960802

[94] Gao M, Yi J, Zhu J, Minikes AM, Monian P, Thompson CB, Jiang X. Role of mitochondria in ferroptosis. Mol Cell. 2019;73(2):354–63.e3. doi:10.1016/j.molcel.2018.10.042. 30581146 PMC6338496

[95] Okazaki S, Umene K, Yamasaki J, Suina K, Otsuki Y, Yoshikawa M, Minami Y, Masuko T, Kawaguchi S, Nakayama H, et al. Glutaminolysis-related genes determine sensitivity to xCT-targeted therapy in head and neck squamous cell carcinoma. Cancer Sci. 2019;110(11):3453–3463. doi:10.1111/cas.14182. 31444923 PMC6825010

[96] Fehér J, Élő Á, István L, Nagy ZZ, Radák Z, Scuderi G, Artico M, Kovács I. Microbiota mitochondria disorders as hubs for early age-related macular degeneration. Geroscience. 2022;44(6):2623–2653. doi:10.1007/s11357-022-00620-5. 35978068 PMC9385247

[97] Faria CC, Fortunato RS. The role of dual oxidases in physiology and cancer. Genet Mol Biol. 2020;43(1 suppl. 1):e20190096. doi:10.1590/1678-4685/gmb-2019-0096. 32453337 PMC7265977

[98] Chen X, Li J, Kang R, Klionsky DJ, Tang D. Ferroptosis: machinery and regulation. Autophagy. 2021;17(9):2054–2081. doi:10.1080/15548627.2020.1810918. 32804006 PMC8496712

[99] Herfindal AM, Rocha SDC, Papoutsis D, Bøhn SK, Carlsen H. The ROS-generating enzyme NADPH oxidase 1 modulates the colonic microbiota but offers minor protection against dextran sulfate sodium-induced low-grade colon inflammation in mice. Free Radic Biol Med. 2022;188:298–311. doi:10.1016/j.freeradbiomed.2022.06.234. 35752373

[100] Darby TM, Naudin CR, Luo L, Jones RM. Lactobacillus rhamnosus GG-induced expression of Leptin in the intestine orchestrates epithelial cell proliferation. Cell Mol Gastroenterol Hepatol. 2020;9(4):627–639. doi:10.1016/j.jcmgh.2019.12.004. 31874255 PMC7160578

[101] Grasberger H, Gao J, Nagao-Kitamoto H, Kitamoto S, Zhang M, Kamada N, Eaton KA, El-Zaatari M, Shreiner AB, Merchant JL, et al. Increased expression of DUOX2 is an epithelial response to mucosal dysbiosis required for immune homeostasis in mouse intestine. Gastroenterology. 2015;149(7):1849–1859. doi:10.1053/j.gastro.2015.07.062. 26261005 PMC4663159

[102] Kim SY, Chae CW, Lee HJ, Jung YH, Choi GE, Kim JS, Lim JR, Lee JE, Cho JH, Park H, et al. Sodium butyrate inhibits high cholesterol-induced neuronal amyloidogenesis by modulating NRF2 stabilization-mediated ROS levels: involvement of NOX2 and SOD1. Cell Death Disease. 2020;11(6):469. doi:10.1038/s41419-020-2663-1. 32555166 PMC7303181

[103] Al-Harbi NO, Nadeem A, Ahmad SF, Alotaibi MR, AlAsmari AF, Alanazi WA, Al-Harbi MM, El-Sherbeeny AM, Ibrahim KE. Short chain fatty acid, acetate ameliorates sepsis-induced acute kidney injury by inhibition of NADPH oxidase signaling in T cells. Int Immunopharmacol. 2018;58:24–31. doi:10.1016/j.intimp.2018.02.023. 29544198

[104] Carroll IM, Andrus JM, Bruno-Bárcena JM, Klaenhammer TR, Hassan HM, Threadgill DS. Anti-inflammatory properties of Lactobacillus gasseri expressing manganese superoxide dismutase using the interleukin 10-deficient mouse model of colitis. Am J Physiol Gastrointest Liver Physiol. 2007;293(4):G729–38. doi:10.1152/ajpgi.00132.2007. 17640978

[105] Yin H, Xu L, Porter NA. Free radical lipid peroxidation: mechanisms and analysis. Chem Rev. 2011;111(10):5944–5972. doi:10.1021/cr200084z. 21861450

[106] Liang D, Minikes AM, Jiang X. Ferroptosis at the intersection of lipid metabolism and cellular signaling. Mol Cell. 2022;82(12):2215–2227. doi:10.1016/j.molcel.2022.03.022. 35390277 PMC9233073

[107] Zou Y, Palte MJ, Deik AA, Li H, Eaton JK, Wang W, Tseng YY, Deasy R, Kost-Alimova M, Dančík V, et al. A GPX4-dependent cancer cell state underlies the clear-cell morphology and confers sensitivity to ferroptosis. Nat Commun. 2019;10(1):1617. doi:10.1038/s41467-019-09277-9. 30962421 PMC6453886

[108] Aron-Wisnewsky J, Warmbrunn MV, Nieuwdorp M, Clément K. Metabolism and metabolic disorders and the microbiome: the intestinal microbiota associated with obesity, lipid metabolism, and metabolic health-pathophysiology and therapeutic strategies. Gastroenterology. 2021;160(2):573–599. doi:10.1053/j.gastro.2020.10.057. 33253685

[109] Schoeler M, Caesar R. Dietary lipids, gut microbiota and lipid metabolism. Rev Endocr Metab Disord. 2019;20(4):461–472. doi:10.1007/s11154-019-09512-0. 31707624 PMC6938793

[110] Abedi E, Sahari MA. Long-chain polyunsaturated fatty acid sources and evaluation of their nutritional and functional properties. Food Sci Nutr. 2014;2(5):443–463. doi:10.1002/fsn3.121. 25473503 PMC4237475

[111] Miyamoto J, Igarashi M, Watanabe K, Karaki SI, Mukouyama H, Kishino S, Li X, Ichimura A, Irie J, Sugimoto Y, et al. Gut microbiota confers host resistance to obesity by metabolizing dietary polyunsaturated fatty acids. Nat Commun. 2019;10(1):4007. doi:10.1038/s41467-019-11978-0. 31488836 PMC6728375

[112] Fu Y, Wang Y, Gao H, Li D, Jiang R, Ge L, Tong C, Xu K. Associations among dietary omega-3 polyunsaturated fatty acids, the gut microbiota, and intestinal immunity. Mediators Inflamm. 2021;2021(8879227):1–11. doi:10.1155/2021/8879227. PMC7801035 33488295

[113] Kagan VE, Mao G, Qu F, Angeli JP, Doll S, Croix CS, Dar HH, Liu B, Tyurin VA, Ritov VB, et al. Oxidized arachidonic and adrenic PEs navigate cells to ferroptosis. Nat Chem Biol. 2017;13(1):81–90. doi:10.1038/nchembio.2238. 27842066 PMC5506843

[114] Doll S, Conrad M. Iron and ferroptosis: a still ill-defined liaison. Iubmb Life. 2017;69(6):423–434. doi:10.1002/iub.1616. 28276141

[115] Liang C, Zhou XH, Gong PM, Niu HY, Lyu LZ, Wu YF, Han X, Zhang L-W. Lactiplantibacillus plantarum H-87 prevents high-fat diet-induced obesity by regulating bile acid metabolism in C57BL/6J mice. Food Funct. 2021;12(10):4315–4324. doi:10.1039/D1FO00260K. 34031676

[116] Ning K, Lu K, Chen Q, Guo Z, Du X, Riaz F, Feng L, Fu Y, Yin C, Zhang F, et al. Epigallocatechin gallate protects mice against methionine–choline-Deficient-Diet-Induced nonalcoholic steatohepatitis by improving gut microbiota to attenuate hepatic injury and regulate metabolism. ACS Omega. 2020;5(33):20800–20809. doi:10.1021/acsomega.0c01689. 32875214 PMC7450495

[117] Foret MK, Lincoln R, Do Carmo S, Cuello AC, Cosa G. Connecting the “dots”: from free radical lipid autoxidation to cell pathology and disease. Chem Rev. 2020;120(23):12757–12787. doi:10.1021/acs.chemrev.0c00761. 33211489

[118] Tang D, Kroemer G. Ferroptosis. Curr Biol. 2020;30(21):R1292–R1297. doi:10.1016/j.cub.2020.09.068. 33142092

[119] Koppula P, Zhang Y, Zhuang L, Gan B. Amino acid transporter SLC7A11/xCT at the crossroads of regulating redox homeostasis and nutrient dependency of cancer. Cancer Commun (Lond). 2018;38(1):1–13. doi:10.1186/s40880-018-0288-x. 29764521 PMC5993148

[120] Jiang L, Kon N, Li T, Wang SJ, Su T, Hibshoosh H, Baer R, Gu W. Ferroptosis as a p53-mediated activity during tumour suppression. Nature. 2015;520(7545):57–62. doi:10.1038/nature14344. 25799988 PMC4455927

[121] Kong B, Fu H, Xiao Z, Zhou Y, Shuai W, Huang H. Gut microbiota dysbiosis induced by a high-fat diet increases susceptibility to atrial fibrillation. Can J Cardiol. 2022;38(12):1962–1975. doi:10.1016/j.cjca.2022.08.231. 36084771

[122] Huang C, Wu D, Zhang K, Khan FA, Pandupuspitasari NS, Wang Y, Huo L, Sun F. Perfluorooctanoic acid alters the developmental trajectory of female germ cells and embryos in rodents and its potential mechanism. Ecotoxicol Environ Saf. 2022;236:113467. doi:10.1016/j.ecoenv.2022.113467. 35390687

[123] Bersuker K, Hendricks JM, Li Z, Magtanong L, Ford B, Tang PH, Roberts MA, Tong B, Maimone TJ, Zoncu R, et al. The CoQ oxidoreductase FSP1 acts parallel to GPX4 to inhibit ferroptosis. Nature. 2019;575(7784):688–692. doi:10.1038/s41586-019-1705-2. 31634900 PMC6883167

[124] Doll S, Freitas FP, Shah R, Aldrovandi M, da Silva MC, Ingold I, Goya Grocin A, Xavier da Silva TN, Panzilius E, Scheel CH, et al. FSP1 is a glutathione-independent ferroptosis suppressor. Nature. 2019;575(7784):693–698. doi:10.1038/s41586-019-1707-0. 31634899

[125] Viswanathan VS, Ryan MJ, Dhruv HD, Gill S, Eichhoff OM, Seashore-Ludlow B, Kaffenberger SD, Eaton JK, Shimada K, Aguirre AJ, et al. Dependency of a therapy-resistant state of cancer cells on a lipid peroxidase pathway. Nature. 2017;547(7664):453–457. doi:10.1038/nature23007. 28678785 PMC5667900

[126] Ma C, Wu X, Zhang X, Liu X, Deng G. Heme oxygenase-1 modulates ferroptosis by fine-tuning levels of intracellular iron and reactive oxygen species of macrophages in response to Bacillus Calmette-Guerin infection. Front Cell Infect Microbiol. 2022;12:1004148. doi:10.3389/fcimb.2022.1004148. 36211962 PMC9539760

[127] Yue M, Wei J, Chen W, Hong D, Chen T, Fang X. Neurotrophic role of the Next-generation probiotic Strain L. lactis MG1363-pMg36e-GLP-1 on Parkinson’s disease via inhibiting ferroptosis. Nutrients. 2022;14(22):4886. doi:10.3390/nu14224886. 36432569 PMC9698534

[128] Kraft VAN, Bezjian CT, Pfeiffer S, Ringelstetter L, Müller C, Zandkarimi F, Merl-Pham J, Bao X, Anastasov N, Kössl J, et al. GTP cyclohydrolase 1/Tetrahydrobiopterin counteract ferroptosis through lipid remodeling. ACS Cent Sci. 2020;6(1):41–53. doi:10.1021/acscentsci.9b01063. 31989025 PMC6978838

[129] Soula M, Weber RA, Zilka O, Alwaseem H, La K, Yen F, Molina H, Garcia-Bermudez J, Pratt DA, Birsoy K, et al. Metabolic determinants of cancer cell sensitivity to canonical ferroptosis inducers. Nat Chem Biol. 2020;16(12):1351–1360. doi:10.1038/s41589-020-0613-y. 32778843 PMC8299533

[130] Gangula P, Ravella K, Chukkapalli S, Rivera M, Srinivasan S, Hale A, Channon K, Southerland J, Kesavalu L. Polybacterial periodontal pathogens alter vascular and gut BH4/nNOS/NRF2-phase II enzyme expression. PloS One. 2015;10(6):e0129885. doi:10.1371/journal.pone.0129885. 26111153 PMC4482323

[131] Shanmuganathan MV, Krishnan S, Fu X, Prasadarao NV. Escherichia coli K1 induces pterin production for enhanced expression of Fcγ receptor I to invade RAW 264.7 macrophages. Microbes Infect. 2014;16(2):134–141. doi:10.1016/j.micinf.2013.10.013. 24161960 PMC3946618

[132] Melo FHM, Gonçalves DA, Sousa RX, Icimoto MY, Fernandes DC, Laurindo FRM, Jasiulionis MG. Metastatic melanoma progression is associated with endothelial nitric oxide synthase uncoupling induced by loss of eNOS: BH4 stoichiometry. Int J Mol Sci. 2021;22(17):9556. doi:10.3390/ijms22179556. 34502464 PMC8430733

[133] Toyokuni S, Ito F, Yamashita K, Okazaki Y, Akatsuka S. Iron and thiol redox signaling in cancer: an exquisite balance to escape ferroptosis. Free Radic Biol Med. 2017;108:610–626. doi:10.1016/j.freeradbiomed.2017.04.024. 28433662

[134] Telorack M, Meyer M, Ingold I, Conrad M, Bloch W, Werner S, Yuspa SH. A glutathione-Nrf2-thioredoxin cross-talk ensures keratinocyte survival and efficient wound repair. PLoS Genet. 2016;12(1):e1005800. doi:10.1371/journal.pgen.1005800. 26808544 PMC4726503

[135] Balsera M, Buchanan BB. Evolution of the thioredoxin system as a step enabling adaptation to oxidative stress. Free Radic Biol Med. 2019;140:28–35. doi:10.1016/j.freeradbiomed.2019.03.003. 30862542

[136] Zhang Y, Ma J, Jing N, Zhang H, Xie Y, Liu H, Shan X, Ren J, Jin J. Bifidobacterium animalis A12 and Lactobacillus salivarius M18-6 alleviate alcohol injury by keap1-Nrf2 pathway and thioredoxin system. Foods. 2023;12(3):439. doi:10.3390/foods12030439. 36765968 PMC9914461

[137] Gan F, Chen X, Liao SF, Lv C, Ren F, Ye G, Pan C, Huang D, Shi J, Shi X, et al. Selenium-enriched probiotics improve antioxidant status, immune function, and selenoprotein gene expression of piglets raised under high ambient temperature. J Agric Food Chem. 2014;62(20):4502–4508. doi:10.1021/jf501065d. 24814575

[138] Kapralov AA, Yang Q, Dar HH, Tyurina YY, Anthonymuthu TS, Kim R, St Croix CM, Mikulska-Ruminska K, Liu B, Shrivastava IH, et al. Redox lipid reprogramming commands susceptibility of macrophages and microglia to ferroptotic death. Nat Chem Biol. 2020;16(3):278–290. doi:10.1038/s41589-019-0462-8. 32080625 PMC7233108

[139] Radi R. Peroxynitrite, a stealthy biological oxidant. J Biol Chem. 2013;288(37):26464–26472. doi:10.1074/jbc.R113.472936. 23861390 PMC3772193

[140] Rubbo H, Parthasarathy S, Barnes S, Kirk M, Kalyanaraman B, Freeman BA. Nitric oxide inhibition of lipoxygenase-dependent liposome and low-density lipoprotein oxidation: termination of radical chain propagation reactions and formation of nitrogen-containing oxidized lipid derivatives. Arch Biochem Biophys. 1995;324(1):15–25. doi:10.1006/abbi.1995.9935. 7503550

[141] Kuhn H, Saam J, Eibach S, Holzhütter HG, Ivanov I, Walther M. Structural biology of mammalian lipoxygenases: enzymatic consequences of targeted alterations of the protein structure. Biochem Biophys Res Commun. 2005;338(1):93–101. doi:10.1016/j.bbrc.2005.08.238. 16168952

[142] Yan B, Ai Y, Sun Q, Ma Y, Cao Y, Wang J, Zhang Z, Wang X. Membrane damage during ferroptosis is caused by oxidation of phospholipids catalyzed by the oxidoreductases POR and CYB5R1. Mol Cell. 2021;81(2):355–69.e10. doi:10.1016/j.molcel.2020.11.024. 33321093

[143] Zou Y, Li H, Graham ET, Deik AA, Eaton JK, Wang W, Sandoval-Gomez G, Clish CB, Doench JG, Schreiber SL, et al. Cytochrome P450 oxidoreductase contributes to phospholipid peroxidation in ferroptosis. Nat Chem Biol. 2020;16(3):302–309. doi:10.1038/s41589-020-0472-6. 32080622 PMC7353921

[144] Çolakoğlu M, Tunçer S, Banerjee S. Emerging cellular functions of the lipid metabolizing enzyme 15-lipoxygenase-1. Cell Prolif. 2018;51(5):e12472. doi:10.1111/cpr.12472. 30062726 PMC6528901

[145] Mathis SP, Bodduluri SR, Haribabu B. Interrelationship between the 5-lipoxygenase pathway and microbial dysbiosis in the progression of Alzheimer’s disease. Biochim Biophys Acta Mol Cell Biol Lipids. 2021;1866(9):158982. doi:10.1016/j.bbalip.2021.158982. 34062254 PMC11522975

[146] Grabauskas G, Gao J, Wu X, Zhou SY, Turgeon DK, Owyang C. WITHDRAWN: gut microbiota alter visceral pain sensation and inflammation via modulation of synthesis of resolvin D1 in colonic Tuft cells. Gastroenterology. 2022. doi:10.1053/j.gastro.2022.07.053. PMC9898459 35934059

[147] Villarreal-Soto SA, Bouajila J, Pace M, Leech J, Cotter PD, Souchard JP, Taillandier P, Beaufort S. Metabolome-microbiome signatures in the fermented beverage, Kombucha. Int J Food Microbiol. 2020;333(108778):108778. doi:10.1016/j.ijfoodmicro.2020.108778. 32731153

[148] Dar HH, Anthonymuthu TS, Ponomareva LA, Souryavong AB, Shurin GV, Kapralov AO, Tyurin VA, Lee JS, Mallampalli RK, Wenzel SE, et al. A new thiol-independent mechanism of epithelial host defense against Pseudomonas aeruginosa: iNOS/NO• sabotage of theft-ferroptosis. Redox Biol. 2021;45:102045. doi:10.1016/j.redox.2021.102045. 34167028 PMC8227829

[149] Guan Z, Jin X, Guan Z, Liu S, Tao K, Luo L. The gut microbiota metabolite capsiate regulate SLC2A1 expression by targeting HIF-1α to inhibit knee osteoarthritis-induced ferroptosis. Aging Cell. 2023;22(6):e13807. doi:10.1111/acel.13807. 36890785 PMC10265160

[150] Lee EJ, Jeon MS, Kim BD, Kim JH, Kwon YG, Lee H, Lee YS, Yang J-H, Kim T-Y. Capsiate inhibits ultraviolet B-induced skin inflammation by inhibiting src family kinases and epidermal growth factor receptor signaling. Free Radic Biol Med. 2010;48(9):1133–1143. doi:10.1016/j.freeradbiomed.2010.01.034. 20123015

[151] Zeng Y, Wu R, Wang F, Li S, Li L, Li Y, Qin P, Wei M, Yang J, Wu J, et al. Liberation of daidzein by gut microbial β-galactosidase suppresses acetaminophen-induced hepatotoxicity in mice. Cell Host & Microbe. 2023;31(5):766–80.e7. doi:10.1016/j.chom.2023.04.002. 37100057

[152] Wang X, Chen X, Zhou W, Men H, Bao T, Sun Y, Wang Q, Tan Y, Keller BB, Tong Q, et al. Ferroptosis is essential for diabetic cardiomyopathy and is prevented by sulforaphane via AMPK/NRF2 pathways. Acta Pharm Sin B. 2022;12(2):708–722. doi:10.1016/j.apsb.2021.10.005. 35256941 PMC8897044

[153] Li X, Zou Y, Xing J, Fu YY, Wang KY, Wan PZ, Zhai XY. Pretreatment with Roxadustat (FG-4592) attenuates folic acid-induced kidney injury through antiferroptosis via Akt/GSK-3β/Nrf2 pathway. Oxid Med Cell Longev. 2020;2020(6286984):1–17. doi:10.1155/2020/6286984. PMC6995323 32051732

[154] Brenner H, Kloor M, Pox CP. Colorectal cancer. Lancet. 2014;383(9927):1490–1502. doi:10.1016/S0140-6736(13)61649-9. 24225001

[155] Kumar A, Ali A, Kapardar RK, Dar GM, Nimisha A, Sharma AK, Verma R, Sattar RSA, Ahmad E, Mahajan B, et al. Implication of gut microbes and its metabolites in colorectal cancer. J Cancer Res Clin Oncol. 2023;149(1):441–465. doi:10.1007/s00432-022-04422-2. 36572792 PMC11798267

[156] Xu S, He Y, Lin L, Chen P, Chen M, Zhang S. The emerging role of ferroptosis in intestinal disease. Cell Death Disease. 2021;12(4):289. doi:10.1038/s41419-021-03559-1. 33731703 PMC7969743

[157] Wang Y, Zhang Z, Sun W, Zhang J, Xu Q, Zhou X, Mao L. Ferroptosis in colorectal cancer: potential mechanisms and effective therapeutic targets. Biomed Pharmacother. 2022;153(113524):113524. doi:10.1016/j.biopha.2022.113524. 36076606

[158] Li Y, Feng D, Wang Z, Zhao Y, Sun R, Tian D, Liu D, Zhang F, Ning S, Yao J, et al. Ischemia-induced ACSL4 activation contributes to ferroptosis-mediated tissue injury in intestinal ischemia/reperfusion. Cell Death Differ. 2019;26(11):2284–2299. doi:10.1038/s41418-019-0299-4. 30737476 PMC6889315

[159] Zhang X, Wu J, Liu Q, Li X, Li S, Chen J, Hong Z, Wu X, Zhao Y, Ren J, et al. mtDNA-STING pathway promotes necroptosis-dependent enterocyte injury in intestinal ischemia reperfusion. Cell Death Disease. 2020;11(12):1050. doi:10.1038/s41419-020-03239-6. 33311495 PMC7732985

[160] Hu Y, Mao Z, Xu L, Yin L, Tao X, Tang Z, Qi Y, Sun P, Peng J. Protective effect of dioscin against intestinal ischemia/reperfusion injury via adjusting miR-351-5p-mediated oxidative stress. Pharmacol Res. 2018;137:56–63. doi:10.1016/j.phrs.2018.09.016. 30240824

[161] Ozkan OV, Yuzbasioglu MF, Ciralik H, Kurutas EB, Yonden Z, Aydin M, Bulbuloglu E, Semerci E, Goksu M, Atli Y, et al. Resveratrol, a natural antioxidant, attenuates intestinal ischemia/reperfusion injury in rats. Tohoku J Exp Med. 2009;218(3):251–258. doi:10.1620/tjem.218.251. 19561396

[162] Balogh N, Krausz F, Lévai P, Ribiczeyné PS, Vajdovich P, Gaál T. Effect of deferoxamine and L-arginine treatment on lipid peroxidation in an intestinal ischaemia-reperfusion model in rats. Acta Vet Hung. 2002;50(3):343–356. doi:10.1556/avet.50.2002.3.10. 12237975

[163] Fabián O, Kamaradová K. Morphology of inflammatory bowel diseases (IBD). Cesk Patol. 2022;58:27–37. 35387455

[164] Xu M, Tao J, Yang Y, Tan S, Liu H, Jiang J, Zheng F, Wu B. Ferroptosis involves in intestinal epithelial cell death in ulcerative colitis. Cell Death Disease. 2020;11(2):86. doi:10.1038/s41419-020-2299-1. 32015337 PMC6997394

[165] Millar AD, Rampton DS, Blake DR. Effects of iron and iron chelation in vitro on mucosal oxidant activity in ulcerative colitis. Aliment Pharmacol Ther. 2000;14(9):1163–1168. doi:10.1046/j.1365-2036.2000.00828.x. 10971233

[166] Kinchen J, Chen HH, Parikh K, Antanaviciute A, Jagielowicz M, Fawkner-Corbett D, Ashley N, Cubitt L, Mellado-Gomez E, Attar M, et al. Structural remodeling of the human colonic mesenchyme in inflammatory bowel disease. Cell. 2018;175(2):372–86.e17. doi:10.1016/j.cell.2018.08.067. 30270042 PMC6176871

[167] Shikata F, Shimada K, Sato H, Ikedo T, Kuwabara A, Furukawa H, Korai M, Kotoda M, Yokosuka K, Makino H, et al. Potential influences of gut microbiota on the formation of intracranial aneurysm. Hypertension. 2019;73(2):491–496. doi:10.1161/HYPERTENSIONAHA.118.11804. 30624992 PMC6530585

[168] Liu Y, Jiao C, Zhang T, Li X, Li P, Lu M, Ye Z, Du Y, Du R, Zhang W, et al. Early-life gut microbiota governs susceptibility to colitis via microbial-derived ether lipids. Research (Wash D C). 2023;6:0037. doi:10.34133/research.0037. 37040489 PMC10076029

[169] Parada Venegas D, De la Fuente MK, Landskron G, González MJ, Quera R, Dijkstra G, Harmsen HJM, Faber KN, Hermoso MA. Short Chain Fatty Acids (SCFAs)-mediated gut epithelial and immune regulation and its relevance for inflammatory bowel diseases. Front Immunol. 2019;10:277. doi:10.3389/fimmu.2019.01486. 30915065 PMC6421268

[170] Singh V, Ahlawat S, Mohan H, Gill SS, Sharma KK. Balancing reactive oxygen species generation by rebooting gut microbiota. J Appl Microbiol. 2022;132(6):4112–4129. doi:10.1111/jam.15504. 35199405

[171] Zhou J, Li M, Chen Q, Li X, Chen L, Dong Z, Zhu W, Yang Y, Liu Z, Chen Q, et al. Programmable probiotics modulate inflammation and gut microbiota for inflammatory bowel disease treatment after effective oral delivery. Nat Commun. 2022;13(1):3432. doi:10.1038/s41467-022-31171-0. 35701435 PMC9198027

[172] Dar HH, Epperly MW, Tyurin VA, Amoscato AA, Anthonymuthu TS, Souryavong AB, Kapralov AA, Shurin GV, Samovich SN, St. Croix CM, et al. P. aeruginosa augments irradiation injury via 15-lipoxygenase–catalyzed generation of 15-HpETE-PE and induction of theft-ferroptosis. JCI Insight. 2022;7(4). doi:10.1172/jci.insight.156013. PMC8876480 35041620

[173] Mahdy MS, Azmy AF, Dishisha T, Mohamed WR, Ahmed KA, Hassan A, Aidy SE, El-Gendy AO. Irinotecan-gut microbiota interactions and the capability of probiotics to mitigate irinotecan-associated toxicity. BMC Microbiol. 2023;23(1):53. doi:10.1186/s12866-023-02791-3. 36864380 PMC9979425

[174] Proneth B, Conrad M. Ferroptosis and necroinflammation, a yet poorly explored link. Cell Death Differ. 2019;26(1):14–24. doi:10.1038/s41418-018-0173-9. 30082768 PMC6294786

[175] Kontoghiorghes GJ, Neocleous K, Kolnagou A. Benefits and risks of deferiprone in iron overload in thalassaemia and other conditions: comparison of epidemiological and therapeutic aspects with deferoxamine. Drug Saf. 2003;26(8):553–584. doi:10.2165/00002018-200326080-00003. 12825969

[176] Kontoghiorghe CN, Kolnagou A, Kontoghiorghes GJ. Potential clinical applications of chelating drugs in diseases targeting transferrin-bound iron and other metals. Expert Opin Investig Drugs. 2013;22(5):591–618. doi:10.1517/13543784.2013.787408. 23586878

[177] Moreau C, Danel V, Devedjian JC, Grolez G, Timmerman K, Laloux C, Petrault M, Gouel F, Jonneaux A, Dutheil M, et al. Could conservative iron chelation lead to neuroprotection in amyotrophic lateral sclerosis? Antioxid Redox Signal. 2018;29(8):742–748. doi:10.1089/ars.2017.7493. 29287521 PMC6067092

[178] Devos D, Moreau C, Devedjian JC, Kluza J, Petrault M, Laloux C, Jonneaux A, Ryckewaert G, Garçon G, Rouaix N, et al. Targeting chelatable iron as a therapeutic modality in Parkinson’s disease. Antioxid Redox Signal. 2014;21(2):195–210. doi:10.1089/ars.2013.5593. 24251381 PMC4060813

[179] Martin-Bastida A, Ward RJ, Newbould R, Piccini P, Sharp D, Kabba C, Patel MC, Spino M, Connelly J, Tricta F, et al. Brain iron chelation by deferiprone in a phase 2 randomised double-blinded placebo controlled clinical trial in Parkinson’s disease. Sci Rep. 2017;7(1):1398. doi:10.1038/s41598-017-01402-2. 28469157 PMC5431100

